# Effects of table tennis on vision in children and adolescents: a multilevel dose–response meta-analysis

**DOI:** 10.3389/fmed.2026.1829768

**Published:** 2026-05-28

**Authors:** Xin Jin, Ying Wang, Jianwei Guo, Aibin Cao

**Affiliations:** 1 School of Physical Education, Shanxi University, Taiyuan, Shanxi, China; 2 Shanghai University of Sport, Shanghai, China

**Keywords:** adolescents, children, dose–response relationship, multilevel meta-analysis, randomized controlled trials, table tennis, vision

## Abstract

**Objective:**

To evaluate the effects of table tennis exercise on uncorrected visual acuity-related outcomes in children and adolescents and to assess the dose–effect relationship within a dose–response framework.

**Methods:**

In accordance with PRISMA guidelines and PROSPERO registration, we searched PubMed, Web of Science, PsycINFO, Cochrane Library, and CNKI for studies published through February 11, 2026. Eligible studies were randomized controlled trials (RCTs) in Chinese or English involving children or adolescents, comparing table tennis exercise with care, no intervention, or non-table tennis controls, and reporting visual outcomes assessed with visual acuity tools. We excluded non-original, non-experimental, preclinical, and review studies; meta-analyses; conference abstracts; gray literature; duplicate publications; unavailable full texts; and consultation interventions. A three-level random-effects model (REML) was used to pool effect sizes (Hedges’ g), and analyses of heterogeneity, publication bias, sensitivity, subgroup effects, and dose–response relationships were conducted using cubic splines.

**Results:**

This study included nine RCTs, comprising 32 effect sizes from 531 participants. After outlier removal, 31 effect sizes were retained for analysis. Five studies had a risk of bias. After removing one outlier, table tennis improved vision (SMD = 0.91, 95% CI: 0.42–1.41, *p* < 0.001), despite heterogeneity (I^2^ = 85.5%). No evidence of publication bias was detected. Subgroup analyses indicated effects for twice-weekly training (SMD = 1.91) and for adolescents (SMD = 1.46). Dose–response analysis showed a pattern, with results observed at approximately 2 sessions per week, 40 min per session, over approximately 15 weeks. The model estimated an optimal total dose of 19.8–22.8 h, corresponding to an SMD of approximately 0.86. This finding should be regarded as exploratory evidence and interpreted with caution.

**Conclusion:**

Table tennis exercise may improve uncorrected visual acuity-related outcomes in children and adolescents, but the certainty of the evidence remains low because of high heterogeneity and risk-of-bias concerns. Dose–response evidence suggests that low-to-moderate doses may be most effective, offering valuable guidance for school-based programs. Due to limited reporting of exercise intensity and adherence in the included studies, the “21.3-h” optimal dose proposed here is an exploratory clinical reference, not an absolute standard.

**Systematic review registration:**

The unique identifier is CRD420261334889, which corresponds to the International Prospective Register of Systematic Reviews (PROSPERO). The publicly accessible URL is: https://www.crd.york.ac.uk/prospero/display_record.php?ID=CRD420261334889.

## Introduction

Childhood and adolescence are critical periods for visual development and the adaptability of visual functions. The rise in vision problems and myopia has become a significant global public health concern. Projections suggest that by 2050, around 4.758 billion people worldwide could be affected by myopia (49.8%), including approximately 938 million (9.8%) with high myopia. This trend has serious implications for eye care demand, the risk of complications, and social impact (1). International consensus on myopia research indicates that it affects children’s current learning and daily activities and is associated with a higher risk of severe eye changes and vision loss in adulthood. Consequently, the concepts of “early prevention, delayed onset, and progression control” have become essential in managing lifelong eye health (2, 3). A systematic review and forecasting study on myopia and high myopia among Chinese children and adolescents revealed that the prevalence of myopia increases significantly with age and predicts future trends. This shows that students are a key group for myopia prevention and control efforts (4). From a global perspective on eye health, visual impairment due to refractive error remains one of the leading causes of vision loss. Analyses from the Global Burden of Disease (GBD) study systematically identify the causes and long-term trends of preventable vision impairment, providing a strong evidence base for developing national eye health strategies (5). The Lancet Global Health Commission on Global Eye Health emphasized the importance of integrating eye health into universal health coverage and promoting cross-sector policy coordination to reduce the impact of preventable vision impairment (6).

In light of this context, visual problems among children and adolescents are evident not only in the rising rates of myopia but also in the ongoing presence of visual impairment caused by uncorrected refractive errors. Previous research indicates that multiple factors influence students’ adherence to wearing spectacles. Additionally, limited access to screening, optometric services, and follow-up care has further hampered the success of interventions (7). At the same time, providing systematic vision services in schools has been shown to improve both vision-related outcomes and academic performance. This shows that school-based interventions have significant practical value (8). Regarding risk factors, existing evidence generally indicates that a higher near-work load related to education and insufficient outdoor activity are important modifiable factors contributing to the onset and progression of myopia in children and adolescents (9, 10). Mendelian randomization studies further support a causal link between longer years of education and a higher risk of myopia, while time spent outdoors may partly mediate this relationship (11). Additionally, another study suggested that outdoor time might partly mediate the relationship between education and myopia, thereby strengthening the theoretical pathway through which risk could be reduced during school age via behavioral and environmental changes (12). With the widespread use of digital learning and entertainment, screen viewing has become a new form of visual activity that requires careful management. A recent systematic review and dose–response meta-analysis showed a rate-dependent association between digital screen time and myopia risk, providing quantitative evidence to inform the development of effective screen-time management strategies (13). The reduction in outdoor activities and the shift to home-based learning during the pandemic have been associated with negative changes in refractive status among school-aged children. This suggests that environmental factors significantly influence the development of refractive errors in children (14). These findings collectively highlight the importance of exploring practical, engaging, and sustainable behavioral intervention strategies in school settings.

In response to identified risk factors, the International Myopia Institute (IMI) has summarized myopia control strategies in its intervention white paper, categorizing them into optical, pharmacological, and behavioral/environmental approaches. The paper emphasizes the scalability and synergistic benefits of behavioral interventions at the public health level (15). Its subsequent update further summarizes the evidence progress up to 2024 and emphasizes the importance of thoroughly assessing the effectiveness, adherence, and scalability of interventions (16). Among behavioral interventions, increasing time spent outdoors is among the most well-supported strategies and aligns well with school-based programs. Cluster randomized controlled trials conducted in schools have shown that structured efforts to increase children’s outdoor time can reduce the risk of myopia development or negative refractive changes. Additionally, the effects are closely tied to the duration of the intervention, light exposure, and adherence to the program (17–19). However, in real school settings, simply increasing outdoor time may be limited by academic schedules, space constraints, weather, and supervision costs. This highlights the need to explore alternative or complementary strategies that are practical, engaging, and sustainable.

Table tennis is very accessible and is commonly played in school physical education and extracurricular activities. It involves moderate-intensity exercise alongside significant visual demands, such as rapidly tracking moving targets, frequently shifting focus, and coordinating eye and hand movements. This sport might improve visual function through different mechanisms. On the one hand, engaging in table tennis training could partly replace extended near work and screen time, thereby indirectly reducing the risk of myopia (10, 13).

Current research on how table tennis interventions affect vision in children and adolescents is largely fragmented, relying on small randomized controlled trials conducted in schools. These interventions vary widely in their protocols, especially regarding frequency, session length, and overall duration. Furthermore, many of these studies report data from both eyes or include multiple comparisons within a single study, leading to effect sizes that are inherently hierarchically nested and statistically dependent. In line with our completed research protocol and methodological design, this study aims to strictly include randomized controlled trials, specifically focusing on children and adolescents, and to use standardized visual acuity assessments as the primary outcome measure. Additionally, we will use a multilevel model to address the dependent data structure and adopt a dose–response framework to evaluate the relationship between training dose and effect. Consequently, this study aims to systematically assess the impact of table tennis exercise on visual outcomes in children and adolescents. Based on the pooled effect estimates, we will further examine the quantitative relationships between different components of intervention dosage, such as frequency, session duration, and intervention period, and the observed effects, thus providing more accurate evidence for practical exercise guidelines at the school level.

## Materials and methods

### Study design

In line with the Preferred Reporting Items for Systematic Reviews and Meta-Analyses (PRISMA) guidelines (20), this study thoroughly examined relevant RCTs. To promote transparency, the prespecified protocol was registered with the International Prospective Register of Systematic Reviews (PROSPERO) before beginning the formal literature search and initial screening (registration number: CRD420261334889). All subsequent procedures were strictly adhered to in accordance with the PRISMA statement.

### Study inclusion criteria

The literature screening for this systematic review was conducted using strict criteria. The inclusion criteria were as follows: (1) the study must be an RCT examining the effects of table tennis exercise on vision in children and adolescents; (2) the experimental group must have received at least one documented table tennis intervention, while the control group received no intervention, usual physical education, usual care, or active non-table tennis exercise controls; (3) participants must be children or adolescents, with no restrictions on sex, ethnicity, or social background; (4) the primary outcome must be assessed using a standardized visual acuity scale; and (5) the publication must be written in either Chinese or English, with full-text data available.

The exclusion criteria included non-experimental publications (such as theoretical articles and case reports), preclinical studies (like animal or cell experiments), secondary literature (including meta-analyses and reviews), non-original or unpublished materials (such as conference abstracts, duplicate publications, and grey literature), and unstructured one-time consultation interventions. Additionally, although systematic reviews were not directly included, the research team carefully examined their reference lists to identify potentially eligible original studies.

### Search strategy

To thoroughly identify RCTs examining the effects of table tennis interventions on vision in children and adolescents, this study systematically searched five major databases: PubMed, Web of Science, PsycINFO, the Cochrane Library, and China National Knowledge Infrastructure (CNKI), from their inception until February 11, 2026. The core search framework was built strictly following the PICOS principle: (P) the target population was limited to children and adolescents; (I) the intervention included all table tennis activities conducted at least once; (C) the control group was defined as no intervention, usual care, usual physical education, or active non-table tennis exercise controls; (O) the primary outcome focused on changes in scores on standardized visual acuity assessment scales; and (S) only RCTs were considered eligible evidence. During the implementation stage, the researchers systematically combined controlled vocabulary terms, such as MeSH terms, with free-text terms related to the core concepts of “table tennis,” “children,” “adolescents,” and “vision.” The detailed search strategies for each database are included in the Supplementary material.

### Study selection process

The literature screening and review process in this study fully adhered to PRISMA guidelines. First, all records from major databases were imported into Zotero 7.0 to remove duplicates. Then, two independent reviewers reviewed the titles and abstracts to exclude studies that were initially not relevant to the topic. During this stage, screening decisions were coded as “included” (1) or “excluded” (0), and Cohen’s kappa coefficient was used to measure inter-rater agreement between the two reviewers (21). For studies that passed the initial screening, the research team retrieved the full texts and conducted a detailed selection based on the PICOS framework. When there was disagreement between the two reviewers, a third expert was consulted to resolve the issues through discussion. During the data extraction phase, two researchers independently collected key information, including baseline sample characteristics and intervention specifics, using a predesigned standardized data extraction form. The collected data were then cross-verified for accuracy. Any disagreements were resolved through group discussion within the research team.

### Data synthesis

All statistical analyses in this study were performed using R software (version 4.3.3), primarily utilizing the packages meta, metafor, dosresmeta, rjags, diamet, and ggplot2. For continuous outcomes, effect sizes were calculated based on the consistency of the assessment tools: when the same measurement instrument was used across studies, the mean difference (MD) was pooled; when different scales were used, the SMD was calculated. To reduce bias from small sample sizes, all SMDs were converted to Hedges’ g and adjusted accordingly. The effect size thresholds were defined as follows: g values of 0.2, 0.5, and ≥0.8 indicate small, moderate, and large effects, respectively (22). In this study, the process of extracting participants’ age data focused on the overall mean age reported in the original articles. For studies that only provided an age range, the midpoint of the interval was used as a reasonable estimate. Additionally, when studies reported multiple effect sizes, we extracted data separately for each comparator group and outcome measure. To address potential statistical dependence among these nested effect sizes, we used the rma.mv function to fit a three-level random-effects model in our primary analysis. This method effectively accounts for statistical dependence arising from the nested data structure. The model systematically separates the total variance into three components: sampling error (Level 1), within-study variance (Level 2), and between-study variance (Level 3) (23). During the analysis, the model parameters were estimated using the restricted maximum likelihood (REML) method. To assess potential publication bias, we examined funnel plot symmetry, performed Egger’s regression test, and applied the trim-and-fill method to adjust for any bias identified. Furthermore, this study identified outliers that significantly affected the overall pooled estimates by checking whether the absolute value of the standardized residual exceeded 2.5 and whether Cook’s distance exceeded 3 times its mean (24). To further assess the robustness of the findings, we conducted several sensitivity analyses: (1) a leave-one-out approach was used to evaluate the influence of each study on the overall effect size; (2) meta-regression models were applied using the restricted maximum likelihood (REML) method to investigate the potential moderating effects of intervention duration, population characteristics, and other variables, with bubble plots illustrating the trends of these relationships (25); and (3) subgroup analyses were performed based on study features to compare differences in pooled effect sizes across various conditions. To harmonize outcome measures across different scales, the original data were transformed using the Hedges–Olkin method, defined as follows: $\mathrm{SMD}=\frac{M_{\text{Intervention}}-M_{\text{Control}}}{SD_{\text{Pooled}}}$ , $SD_{\text{Pooled}}=\sqrt{\frac{(n_{1}-1)SD_{1}^{2}+(n_{2}-1)SD_{2}^{2}}{n_{1}+n_{2}-2}}$ (26). In this study, we further developed a hierarchical dose–response model within a Bayesian framework. The model used restricted cubic splines to capture nonlinear trends and employed weakly informative priors along with MCMC algorithms for parameter estimation. This method aimed to improve the accuracy of estimates in small-sample situations or in areas with limited exposure data (27, 28). The results were presented as posterior medians with their 95% credible intervals, and posterior prediction intervals were also included. Additionally, sensitivity analyses were performed to evaluate how the choice of prior distributions and the placement of spline knots influenced the model outcomes.

### Risk of bias (quality) assessment

The quality of the included studies was evaluated using the Cochrane Risk of Bias 2.0 tool for randomized controlled trials (2019 version). This tool addresses five areas: (1) bias from the randomization process; (2) bias caused by deviations from the intended interventions; (3) bias from missing outcome data; (4) bias in outcome measurement; and (5) bias in selecting reported results (29). Two researchers independently assessed the risk of bias in the included studies, rating each of the five domains as “low risk,” “some concerns,” or “high risk.” Any disagreements were resolved through discussion between the reviewers or, if necessary, by arbitration from a third party. Based on this evaluation, we further applied the GRADE framework to assess the overall certainty of evidence for the primary outcomes and the results of each subgroup analysis. The rating process strictly adhered to the predefined downgrading criteria, and the “Summary of Findings” tables were created using the GRADEpro GDT online tool to systematically present the assessment results (30).

## Results

### Study selection

**Figure 1:**
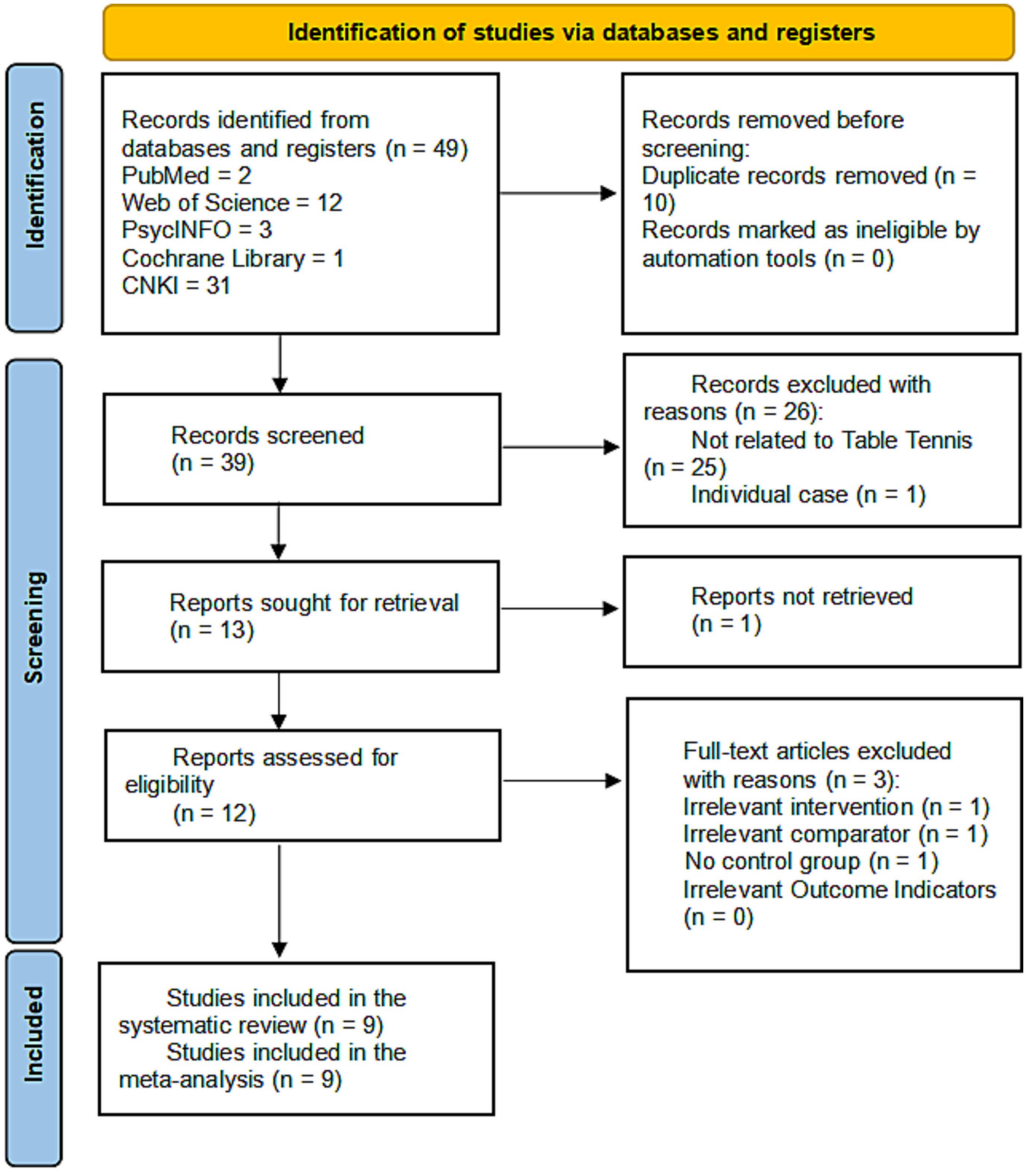
Flow diagram of the selection process.

A total of 49 records were initially identified through a systematic search of relevant databases. After removing duplicates, 10 records were excluded, leaving 39 records for title and abstract screening. Following a thorough review, 26 records were excluded for failing to meet the inclusion criteria, leaving 13 eligible for full-text assessment. Of these, one study was excluded because the full text could not be obtained, and 12 articles were reviewed in depth. As shown in Figure 1, three articles were excluded for specific reasons, leaving nine randomized controlled trials included in the systematic review. All nine studies also met the eligibility criteria for the meta-analysis (31–39).

### Risk of bias of included studies

**Figure 2:**
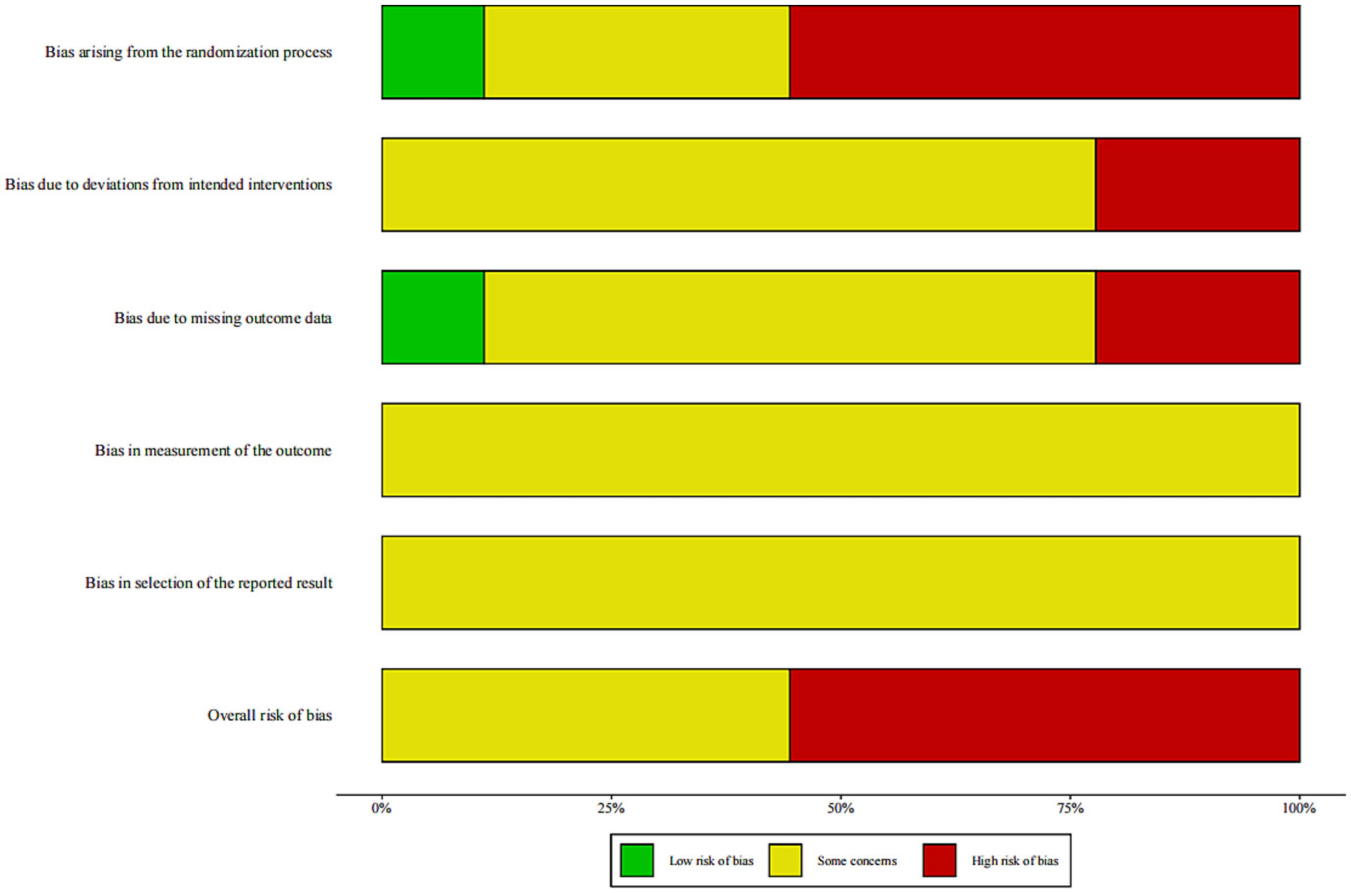
Risk of bias summary.

**Figure 3:**
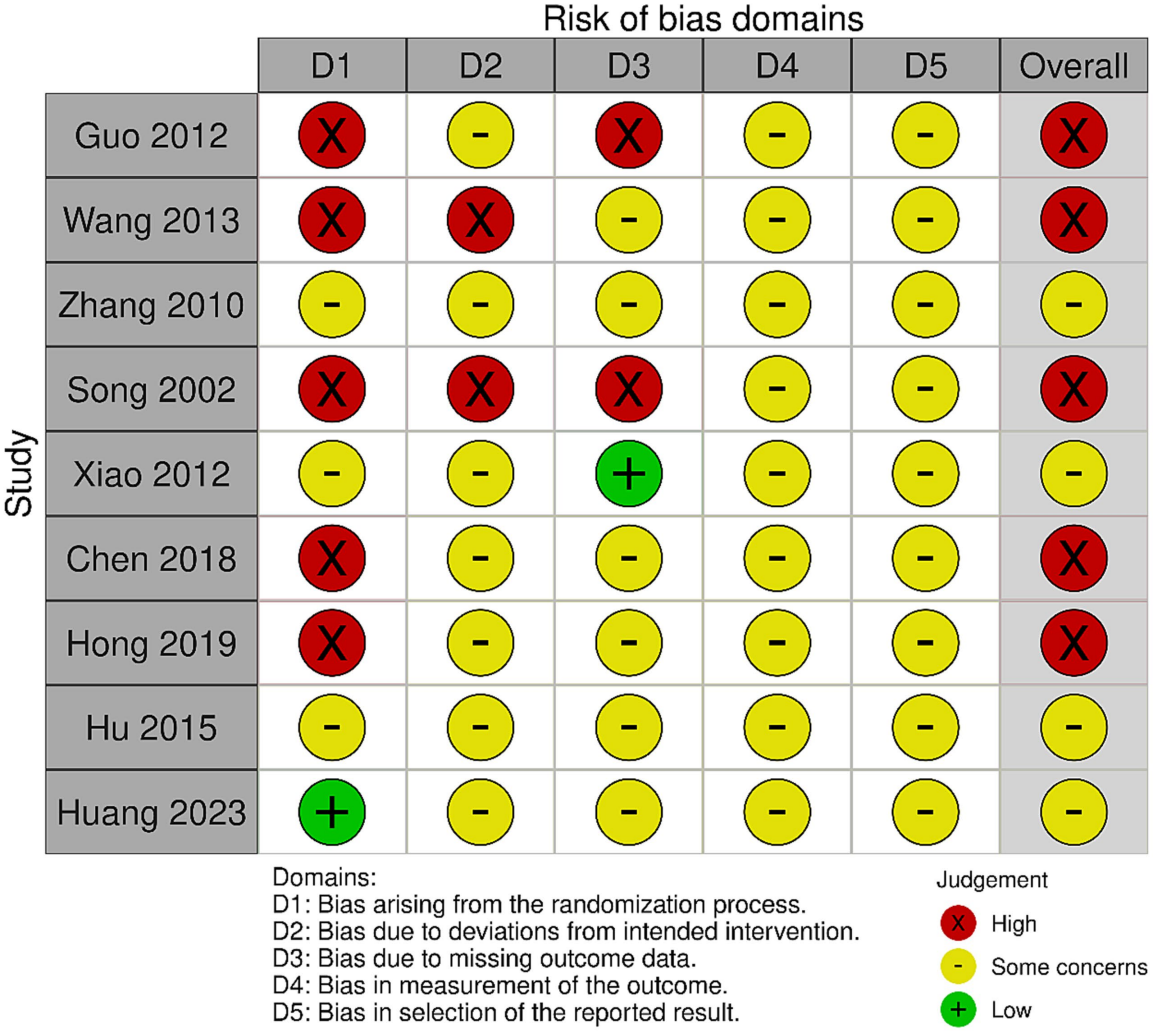
Risk of bias graph.

The risk-of-bias assessment results for the included studies are displayed in Figures 2, 3. Two reviewers independently evaluated the nine included studies across the five domains of RoB 2.0: D1 (bias from the randomization process), D2 (bias due to deviations from the intended interventions), D3 (bias from missing outcome data), D4 (bias in measuring the outcome), and D5 (bias in selecting the reported result). Any disagreements among the reviewers were resolved through discussion to reach a consensus on the final judgments.

The inter-rater agreement results showed that for D1, the simple agreement rate was 66.7% (6/9), with a kappa (*κ*) of 0.481 and a weighted kappa of 0.542, indicating moderate agreement. For D2, the simple agreement rate remained at 66.7% (6/9), while both *κ* and weighted κ were 0.000. In D3, the simple agreement rate was again 66.7% (6/9), with *κ* at 0.413 and weighted κ at 0.491, indicating moderate agreement. For D4, the simple agreement rate increased to 88.9% (8/9), although both κ and weighted κ remained at 0. For D5, full agreement was achieved at 100.0% (9/9), with both κ and weighted κ at 1.000. It is important to note that the “high agreement but low kappa” phenomenon observed in D2 and D4 was mainly due to the prevalence effect, driven by highly concentrated rating distributions.

Regarding bias distribution, the final overall judgments classified five studies as high risk and the remaining four studies as having some concerns. At the domain level, D1 and D2 were identified as the main sources of bias. Several studies showed insufficient reporting on random sequence generation and allocation concealment, with some studies having grouping procedures that were close to post-sampling allocation. Additionally, there was a general lack of information about adherence, contamination control, and the use of intention-to-treat (ITT) analysis. Domain D3 was mostly rated as having some concerns, mainly due to unclear reporting on attrition and how missing data was managed. Domain D4 was also often assessed as having some concerns because information on measurement standardization and assessor blinding was frequently missing. Although the two reviewers fully agreed on D5, most studies in this area still raised concerns due to missing preregistration, protocols, or predefined analysis plans. Because of these issues, we approached the main analysis, the sensitivity analyses, and the evidence quality assessment cautiously, given the potential for systematic errors arising from D1 and D2.

### Study characteristics

The nine studies included in this review were all randomized controlled trials, with sample sizes ranging from 40 to 80 participants. The study populations primarily consisted of children and adolescents aged 6 to 14 years, including both primary and secondary school students. The intervention mainly involved table tennis exercises, with some studies using a three-arm design to compare different types of interventions or to include a usual-care control group. The control conditions mostly involved no intervention, regular physical education classes, or other sports programs. Notable variability was observed in the intensity and duration of the intervention protocols: a single session ranged from 40 to 120 min, with 2 to 4 sessions per week, and the total intervention period ranged from 10 to 96 weeks. Outcomes were primarily assessed using standardized visual evaluation tools, including the Visual Acuity Screening Form (VASF) and the Comprehensive Optometry Instrument (COI). Both tools provided measurement results based on uncorrected visual acuity assessments. Importantly, there was considerable variation among studies due to differences in intervention dosage and outcome definitions (Table 1).

**Table 1:** Characteristics of the studies in the systematic review and meta-analysis.

Author/Year	Country	Design	Sample (T/C)	Age	Subject type	Intervention (T/C)	Protocol (session duration, frequency/week, total duration)	Tools	Eye assessed
Guo et al. (2012) (31)	China	RCT	30/30	12.9	Adolescents	Table Tennis vs. No-intervention control	120 min/session, 3×/week, 96 weeks	VASF	Both eyes
Wang and Liu (2013) (32)	China	RCT	30/30	14.5	Children and Adolescents	Table Tennis vs. Usual physical education class	90 min/session, 3×/week, 24 weeks	VASF	Both eyes
Zhang and Li (2010) (33)	China	RCT	30/30	6.5	Children	Table Tennis vs. Usual physical education class	60 min/session, 4×/week, 10 weeks	VASF	Both eyes
Song et al. (2002) (34)	China	RCT	30/31	10	Children	Table Tennis vs. Usual physical education class	90 min/session, 4×/week, 96 weeks	VASF	Both eyes
Xiao (2012) (35)	China	RCT	30/30	7.5	Children	Table Tennis vs. Middle- and long-distance running training	45 min/session, 3×/week, 16 weeks	VASF	Both eyes
Chen et al. (2018) (36)	China	RCT	30/30	9.5	Children	Table Tennis vs. Usual physical education class	40 min/session, 2×/week, 16 weeks	VASF	Both eyes
Hong (2019) (37)	China	RCT	25/25	10	Children	Table Tennis vs. Usual physical education class	120 min/session, 3×/week, 80 weeks	VASF	Both eyes
Hu (2015) (38)	China	RCT	40/40	10	Children	Table Tennis vs. Basketball training/Middle- and long-distance running training	90 min/session, 4×/week, 32 weeks	VASF	Both eyes
Huang et al. (2023) (39)	China	RCT	20/20	11	Children	Table Tennis vs. Basketball training/Middle- and long-distance running training/Usual physical education class	90 min/session, 3×/week, 24 weeks	COI	Both eyes

RCT denotes a randomized controlled trial. Sample (T/C) indicates the number of participants in the intervention group and control group. The Protocol column reports the session duration, the weekly intervention frequency, and the total intervention duration. Assessment tools include the Visual Acuity Screening Form (VASF) and the Comprehensive Optometry Instrument (COI).

### Multilevel meta-analysis results

**Figure 4:**
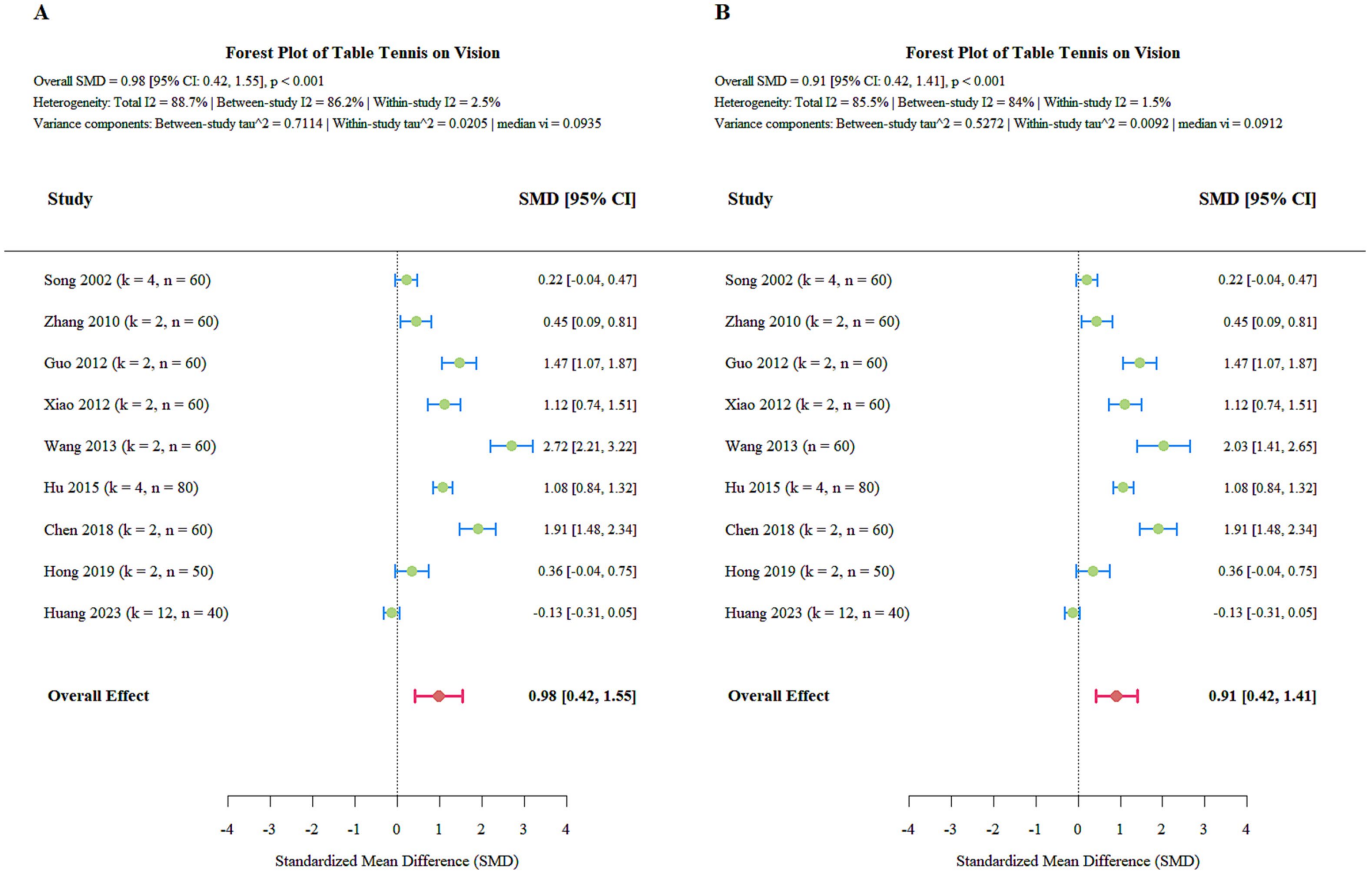
The forest plot shows the effect of table tennis on children’s and adolescents’ visual acuity-related outcomes. Panel **A** shows the pooled-effect forest plot before removal of the outlier, and Panel **B** shows the pooled-effect forest plot after removal of the outlier.

This study systematically evaluated the effect of table tennis exercise on vision in children and adolescents through a meta-analysis of nine randomized controlled trials. Due to the high heterogeneity observed across studies (I^2^ = 88.7%), a random-effects model was used for data synthesis. The meta-analysis indicated that table tennis exercise significantly improved visual outcomes in children and adolescents, with a pooled effect size of SMD = 0.98 (95% CI: 0.42–1.55, *p* = 0.001) (see Figure 4A).

**Figure 5:**
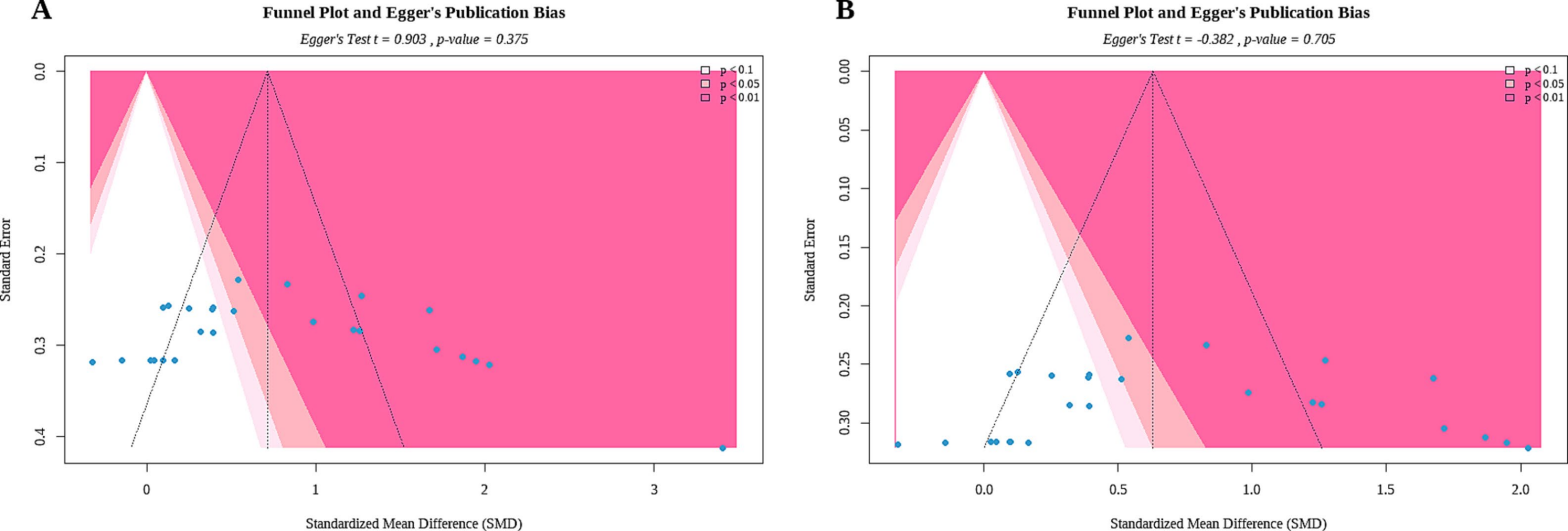
Funnel plots and Egger’s tests for publication bias. Panel **A** shows the funnel plot before removal of the outlier, and Panel **B** shows the funnel plot after removal of the outlier.

**Figure 6:**
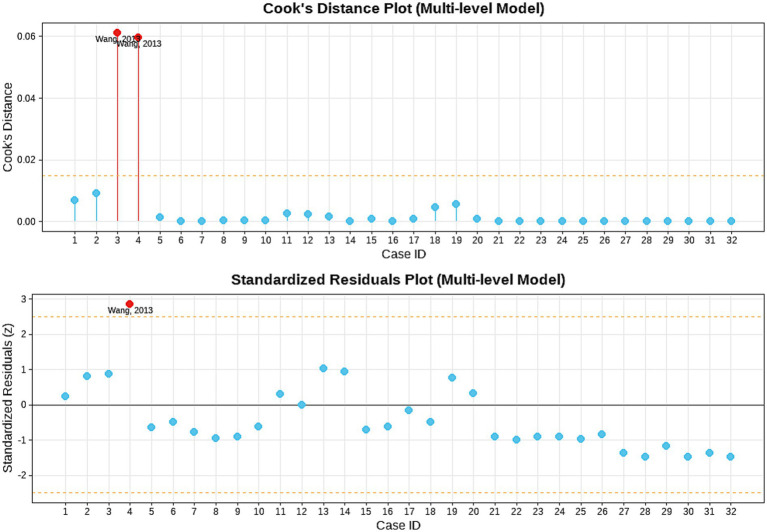
Standardized residuals and Cook’s distance threshold plot (the first time).

To evaluate publication bias and the possible influence of highly influential studies on the overall outcomes, we conducted stepwise diagnostics following the guidelines of the Cochrane Handbook (version 6.3). First, we applied Egger’s regression test to assess the risk of small-study effects (40). The results showed no significant evidence of publication bias regarding the intervention effect (t = 0.903, *p* = 0.375). The corresponding funnel plot is shown in Figure 5A. Next, we used two indicators—standardized residuals (|Z| > 2.5) and Cook’s distance (> three times the mean)—to identify potentially influential studies (24). The results showed that the first dataset from Wang and Liu (32) exceeded the threshold for Cook’s distance; however, because it also failed the criterion for standardized residuals, it was retained. In contrast, the second dataset from Wang and Liu (32) exceeded the thresholds for both indicators. Therefore, it was identified as a highly influential outlier, prompting its removal from the main analysis (Figure 6). During the second round of identification, the first dataset from Wang and Liu (32) did not simultaneously surpass both thresholds and was therefore retained. Further sensitivity analyses showed that the pooled effect size remained generally stable after sequentially removing each study; it increased only when the second dataset from Wang and Liu (32) was excluded, from SMD = 0.98 to 1.06 (see the Supplementary material for details). Additionally, the trim-and-fill analysis indicated that no additional studies were needed, suggesting that publication bias had minimal impact on the results (see the Supplementary material for details).

In summary, after removing one highly influential outlier, this meta-analysis demonstrated that table tennis exercise significantly improved vision in children and adolescents, with a statistically significant pooled effect size (SMD = 0.91, 95% CI: 0.42–1.41, *p* < 0.001) (refer to Figure 4B). Egger’s test showed no significant publication bias (*p* = 0.705; see Figure 5B). To address heterogeneity and potential dependence, a robust variance estimation with small-sample correction (CR2) was performed using the clubSandwich package, producing results consistent with the main analysis (SMD = 0.92, SE = 0.245, t(8.00) = 3.77, *p* = 0.005), thus strengthening the robustness of the findings (see the Supplementary material). According to the GRADE assessment, the certainty of evidence for the primary outcome was rated as low. Two levels downgraded this rating due to the high overall risk of bias in five of the nine included RCTs. At the same time, the remaining four studies raised concerns, particularly regarding randomization processes and deviations from intended interventions. Consequently, despite the pooled effect being statistically significant, the findings should be interpreted with caution. Nonetheless, the main findings demonstrated strong robustness, validated by various diagnostic methods, including Egger’s test, identification and removal of highly influential effects based on standardized residuals and Cook’s distance, robust variance estimation (CR2), sensitivity analyses, and the trim-and-fill method.

### Subgroup analysis

This study conducted prespecified subgroup analyses to systematically identify and quantify potential moderators of the effects of table tennis exercise on vision improvement in children and adolescents. The subgroup variables included: (1) intervention frequency; (2) intervention duration; (3) session length; (4) cumulative intervention dose; (5) participant type; (6) control condition; (7) eye laterality (left vs. right eye); (8) age; (9) baseline refractive status; and (10) baseline refractive error. To examine the role of age, both linear and nonlinear regression models were used to identify potentially complex associations and interactions with treatment outcomes.

**Figure 7:**
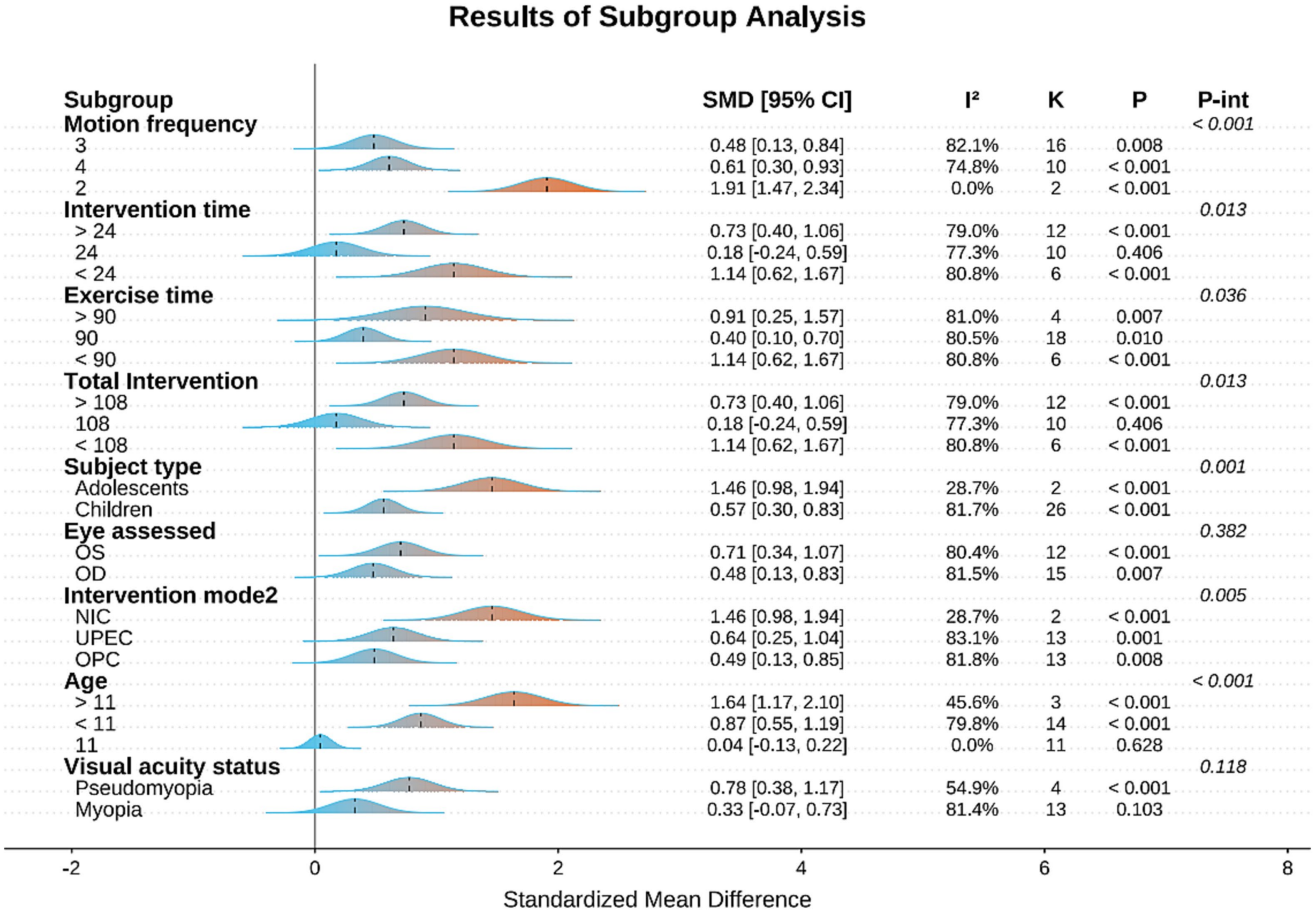
Forest plot of subgroup analysis on the impact of table tennis on the visual acuity-related outcomes of children and adolescents. SMD, standardized mean difference; statistical significance is defined as a 95% confidence interval (95% CI) that does not include zero. *P*-interaction is the *p*-value for tests of subgroup differences. Intervention modes2: NIC, no-intervention control; UPEC, usual physical education class; OPC, outdoor physical activity. Eye assessed: OS, oculus sinister; OD, oculus dexter.

The subgroup results indicated that the benefits of table tennis exercise on vision in children and adolescents varied and were influenced by multiple factors (see Figure 7). Significant differences in interactions were observed across groups concerning intervention parameters (all P-interaction < 0.05). A training frequency of two sessions per week was associated with a larger effect size (SMD = 1.91, 95% CI: 1.47–2.34, *p* < 0.001), while an intervention lasting no more than 24 weeks showed a more significant improvement (SMD = 1.14, 95% CI: 0.62–1.67, *p* < 0.001). Additionally, a session duration of ≤90 min (SMD = 1.14, 95% CI: 0.62–1.67, *p* < 0.001) and a total intervention dose of ≤108 h (SMD = 1.14, 95% CI: 0.62–1.67, *p* < 0.001) were both linked to better outcomes. Regarding participant characteristics, significant differences were also observed by participant type (P-interaction = 0.001), with the adolescent subgroup showing more pronounced benefits (SMD = 1.46, 95% CI: 0.98–1.94, *p* < 0.001). The age subgroup analysis revealed significant differences (P-int < 0.001). Adolescents aged 11 years or older showed a more pronounced benefit (SMD = 1.64, 95% CI: 1.17–2.10, *p* < 0.001), consistent with the observation that older adolescents demonstrated greater improvement. Across control conditions, notable differences were observed across comparator settings (P-int = 0.005). Compared with blank controls, table tennis had the largest effect on vision in children and adolescents (SMD = 1.46, 95% CI: 0.98–1.94, *p* < 0.001), followed by regular physical education classes (SMD = 0.64, 95% CI: 0.25–1.04, *p* = 0.001). However, the sport-specific advantage of table tennis diminished when compared with other outdoor sports (SMD = 0.49, 95% CI: 0.13–0.85, *p* = 0.008). The eye-specific analysis showed that improvement was greater in the left eye than in the right eye: the pooled SMD was 0.71 (95% CI: 0.34–1.07, *p* < 0.001) for the left eye and 0.48 (95% CI: 0.13–0.83, *p* = 0.007) for the right eye. Regarding baseline refractive status, participants with pseudomyopia showed greater improvement (SMD = 0.78, 95% CI: 0.38–1.17, *p* < 0.001). In contrast, participants with myopia showed no significant improvement (SMD = 0.33, 95% CI: −0.07–0.73, *p* = 0.103).

### Linear meta-regression

**Figure 8:**
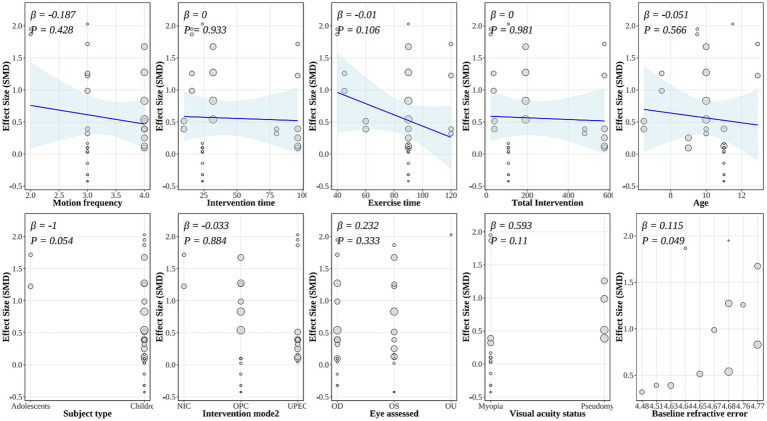
Linear regression bubble chart on the impact of table tennis on the visual acuity-related outcomes of children and adolescents. The solid line represents the fitted linear meta-regression line. The light blue shaded area indicates the 95% confidence interval (CI) around the predicted mean effect. The bubbles represent the estimated effect sizes from individual studies.

The multivariable study-level linear meta-regression analysis (Figure 8) showed that most of the ten specified covariates were not significantly associated with the effect size after adjustment for potential confounders. The covariates included intervention frequency, intervention period, session duration, total intervention dose, age, participant type, control condition, eye laterality, baseline refractive status, and baseline refractive error. Baseline refractive error showed a small positive association with the intervention effect (*β* = 0.115, *p* = 0.049), but this result should be interpreted cautiously given the limited number of studies and the study-level coding of moderators.

### Nonlinear dose–response analysis

**Figure 9:**
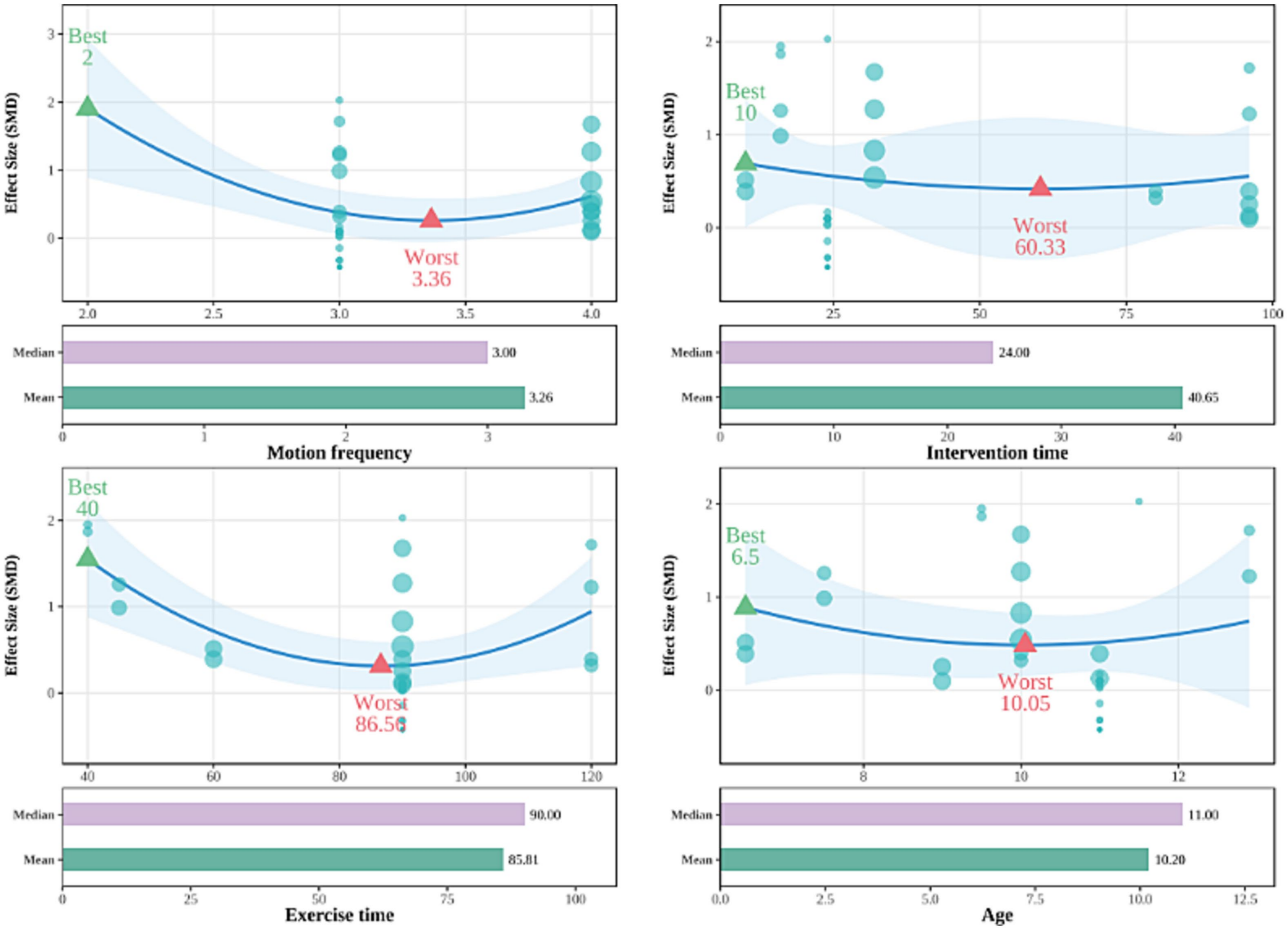
Non-linear regression graph of the impact of table tennis on the visual acuity-related outcomes of children and adolescents.

A clear nonlinear dose–response pattern was observed between the table tennis intervention and visual improvement in children and adolescents. As shown in Figure 9, the effect did not increase steadily with the training dose. Notably, greater improvements were observed even at lower training frequencies, with optimal results occurring around 2 sessions per week, outperforming higher-frequency programs (e.g., 3 sessions per week). Regarding the duration of the intervention, the effect was strongest around ten weeks, indicating that short- to medium-term interventions are likely to produce noticeable visual benefits. In terms of session length, the effect peaked at about 40 min per session; however, as the duration gradually increased to approximately 86 min per session, the estimated effect showed a downward trend, suggesting that overly long sessions may lead to diminishing returns or decreased efficiency. Regarding the age variable, both ends of the nonlinear curve were heavily influenced by a few outlier observations, making them unreliable for clear directional interpretation.

**Figure 10:**
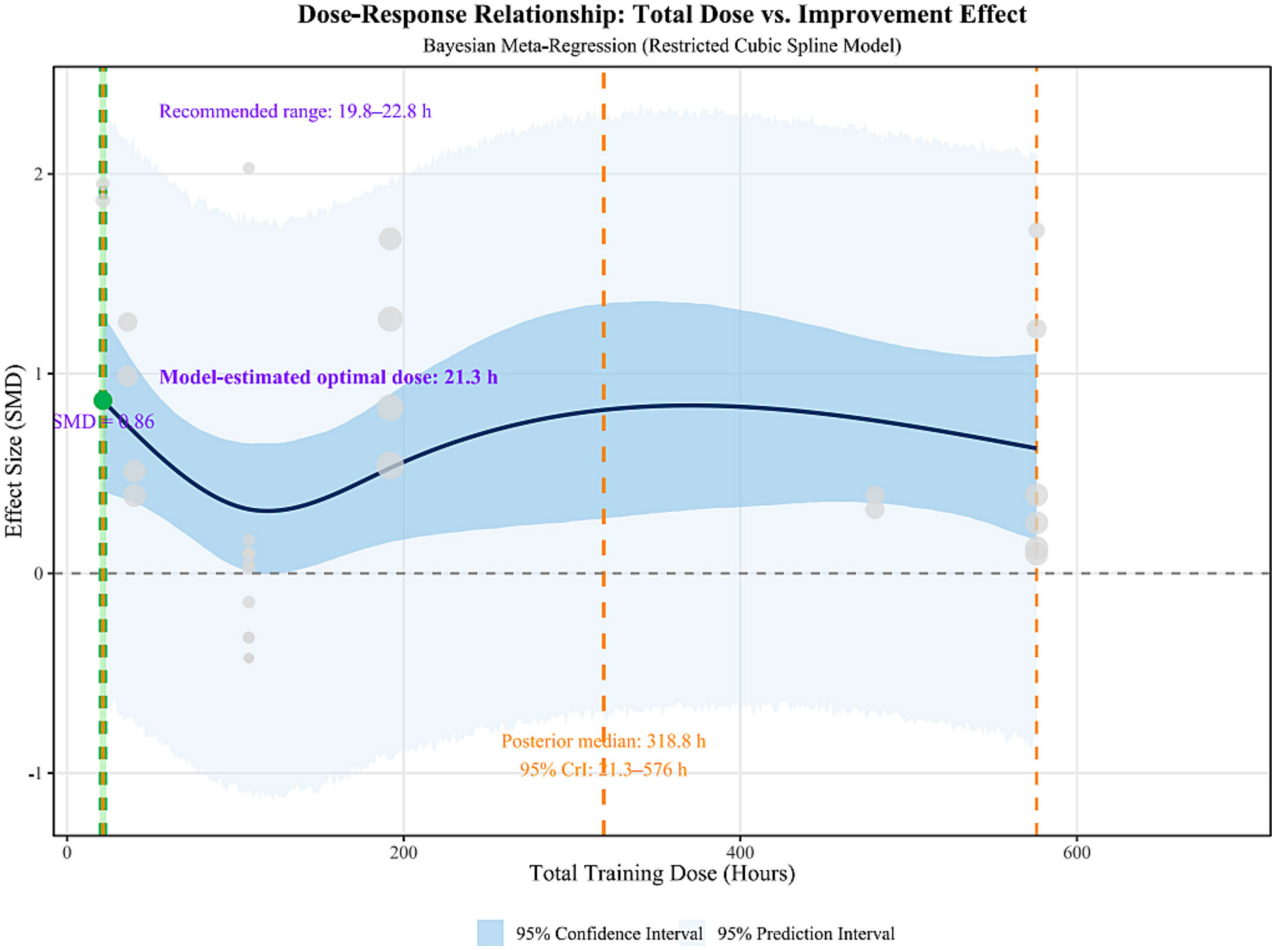
Dose–response relationship graph of the impact of table tennis on the visual acuity-related outcomes of children and adolescents.

The Bayesian dose–response model indicated that the estimated effect peaked at approximately 21.3 h of cumulative exposure to table tennis (Figure 10). A practical dose range near this estimate was found to be 19.8 to 22.8 h, which corresponds to roughly two 40-min sessions per week over 15 to 17 weeks. However, the posterior uncertainty increased at higher cumulative doses, so this estimate should be regarded as exploratory rather than prescriptive.

This study presents an exploratory intervention to enhance vision in children and teens through table tennis exercises. The program includes two 40-min sessions each week over 15 weeks, totaling 19.8–22.8 h. Additionally, this study displays the posterior distribution of the optimal dosage (in hours) derived from posterior sampling. It also features a global density overlay plot that shows the observed effect sizes (yi) alongside the posterior replicated data (yrep), as well as interval plots that illustrate the consistency between the posterior predictive intervals for each observation and the observed values. This study also reported the prior intervals and presented comparative graphs of the prior and posterior intervals. The two types of intervals were generally consistent, indicating a certain degree of stability in the exploratory dose–response analysis. However, due to the limited number of included studies, most of which did not clearly report training intensity, and insufficient monitoring of intervention adherence, the shape of the dose–response curve may have been affected. Consequently, the conclusions of this study should be regarded as exploratory evidence and interpreted cautiously. These visualizations provide a comprehensive assessment of model fit and predictive performance (see the Supplementary material for more details).

## Summary

This systematic review included nine randomized controlled trials mainly involving participants aged 6 to 14 years, including children and adolescents. The intervention protocols for table tennis varied significantly across studies, with session durations ranging from 40 to 120 min, frequencies of 2 to 4 sessions per week, and total intervention periods from 10 to 96 weeks. The multilevel meta-analysis revealed that table tennis exercise significantly improved vision, despite high heterogeneity (I^2^ = 88.7%). After removing one highly influential outlier, the combined effect size was SMD = 0.91 (95% CI: 0.42–1.41, *p* < 0.001). This result remained consistent through multiple robustness checks, indicating a significant overall improvement. Regarding risk of bias, the overall RoB 2.0 assessments classified five studies as high risk and four as having some concerns, with the main risks focused on randomization and deviations from the intended interventions. No significant evidence of publication bias was detected (Egger’s test: t = 0.903, *p* = 0.375), and the trim-and-fill method indicated that no missing studies needed to be added. The leave-one-out sensitivity analysis demonstrated overall robustness; however, excluding the second dataset from Wang and Liu (32) slightly increased the pooled effect size from 0.98 to 1.06. The dose–response analysis revealed a nonlinear pattern of effects, with greater improvements occurring at about 2 sessions per week, lasting around 10 weeks, and each session lasting approximately 40 min. However, the estimated effect decreased as session duration increased to about 86 min, suggesting a limited moderating effect of age. The Bayesian dose–response model indicated that the posterior median cumulative dose was 318.8 h (95% CrI: 21.3–576 h), with the optimal cumulative dose estimated at around 21.3 h (recommended credible interval: 19.8–22.8 h), corresponding to an SMD of approximately 0.86. Therefore, a practical approach of “low-frequency plus moderate-duration” is recommended: specifically, 2 sessions per week, 40 min per session, over approximately 15 weeks. Subgroup analysis further revealed that greater effects were observed with 2 sessions per week, intervention periods not exceeding 24 weeks, session durations of ≤90 min, and cumulative doses of ≤108 h, with adolescents showing greater improvement than children. The main limitations of this analysis included high heterogeneity and a relatively high overall risk of bias in some studies. Future high-quality RCTs should use more standardized protocols and thoroughly report randomization and adherence. Additionally, dose parameters and stratified participant characteristics should be systematically documented within the same study to better identify the optimal dose range and reduce uncertainty.

## Discussion

This study involved nine randomized controlled trials with children and adolescents aged 6 to 14 years. The intervention protocols generally included 2 to 4 sessions per week, each lasting 40 to 120 min, over 10 to 96 weeks. Outcomes were assessed using vision-related tests, such as the VASF or the COI. Although both tools provided uncorrected visual acuity outcomes, they differed in their assessment formats: the VASF was primarily used as a school-based visual acuity screening and recording tool, while the COI served as an instrument-based optometric device with broader examination capabilities. Consequently, only naked-eye visual acuity data were extracted from both tools to enhance comparability across studies. In the multilevel random-effects model, after identifying and removing one highly influential outlier, the pooled effect size for table tennis exercise on visual improvement was an SMD of 0.91 (95% CI: 0.42–1.41, *p* < 0.001). However, overall heterogeneity remained high, with an I^2^ of 85.5%.

Compared to previous evidence, this study’s findings support the broader consensus that behavioral and environmental interventions can improve myopia-related outcomes. Past studies and consensus statements generally indicate that increased near work, limited outdoor activity, insufficient physical activity, and more exposure to digital screens are all associated with a higher risk of myopia or poorer vision outcomes in children and adolescents (10). Systematic reviews on outdoor time also indicate that it offers a protective effect against the development of myopia and refractive changes (41). Additionally, adolescent cohort studies have shown that lower levels of physical activity and increased screen time are linked to a higher risk of myopia (42). Recent dose–response meta-analyses show that greater exposure to digital screens is nonlinearly linked to an increased risk of myopia (13). Most of the external evidence mentioned earlier used myopia-related indicators, such as refractive error, as outcomes. In contrast, the trials included in this study primarily employed vision-related scales. Therefore, the current findings are better interpreted as suggesting that, within the existing trial framework and measurement system, table tennis—defined by high visual demand and moderate-to-vigorous physical activity—may have enhanced visual outcomes by influencing behavioral exposures and visual functional processes related to myopia or visual health.

Based on the theoretical background of accommodation and binocular vision described in the IMI white paper, it is proposed that table tennis exercise may enhance accommodative function during near-work tasks by promoting sustained shifts in focus and visual tracking, thereby positively influencing visual performance (43). Furthermore, if table tennis training partly replaces nearby screen time or increases exposure to natural light, it could lead to additional indirect effects. Reviews and translational studies on dopamine indicate that lighting conditions can influence retinal dopamine signaling, potentially impacting susceptibility to myopia (44). At the same time, short-term changes in indicators such as retinal thickness following outdoor exposure have been reported, suggesting that outdoor environments may protect eye tissue responses (45).

By synthesizing the results of subgroup analyses, nonlinear regression, and Bayesian dose–response modeling, this study suggests that the effects of table tennis exercise on visual improvements in children and adolescents may depend on specific dose thresholds and nonlinear characteristics. Along with previous research, the practicality of intervention programs in school settings, combined with family–school collaboration, seems essential for long-term success (46, 47). For vision-promotion strategies that require long-term implementation, monitoring and maintaining adherence are essential to ensuring the stable translation of intervention effects (48). Therefore, based on the available evidence, this study recommends a program of 2 sessions per week, each lasting 40 min, for approximately 15 weeks as an exploratory intervention in school settings. At the same time, from a potential mechanistic perspective, table tennis exercise may improve visual performance by enhancing eye-tracking ability for moving targets and visual–motor coordination (49). Furthermore, the accommodative function is closely connected to the onset and progression of myopia, and changes in visual activity patterns may also play an important role in its beneficial effects (50). Since both near-work load and light exposure are linked to myopia progression, table tennis exercise may have positive effects by reducing time spent on prolonged near work and, under certain conditions, increasing exposure to higher-illuminance environments (51, 52). Although the prior and posterior intervals in the dose–response analysis were generally consistent, indicating a certain degree of stability, the recommended intervention regimen proposed in this study should still be considered exploratory and interpreted with caution. This caution is primarily due to the limited number of included studies and the potential impact of insufficiently monitored adherence to the intervention on the shape of the dose–response curve. Furthermore, the subgroup analysis revealed that table tennis produced the greatest improvement in vision compared with blank controls, whereas the effect diminished when the control groups participated in regular physical education classes or other outdoor sports. This suggests that the visual benefits of table tennis may partly stem from a time-substitution effect, such as reduced near-work or sedentary activity. Additionally, other physical activities, such as basketball and middle-distance running, may also have the potential to enhance vision in children and adolescents.

From a practical standpoint, table tennis exercises can serve as an effective extra strategy for enhancing visual function in school environments, especially when paired with regular physical activity programs. Emphasizing sustainable, simple-to-implement elements, such as a moderate schedule of 2 sessions per week, each lasting 40 min, is crucial. Future research should focus on conducting high-quality RCTs with larger sample sizes, clear reporting of randomization and allocation concealment, and thorough monitoring of adherence and contamination. Additionally, the definitions of visual outcomes, measurement tools, and assessment time points should be standardized. Prescription-related details, such as frequency, session length, intervention duration, and total dose, must be systematically documented. Additionally, dose stratification or multi-arm comparisons should be established *a priori* within the same study to minimize heterogeneity and enable effective analysis of nonlinear dose ranges and population differences.

## Conclusion

This study included nine randomized controlled trials and used a multilevel random-effects model. After identifying and removing one highly influential outlier, table tennis exercise was significantly associated with improved uncorrected visual acuity-related outcomes in children and adolescents (SMD = 0.91, 95% CI: 0.42–1.41, *p* < 0.001). The sensitivity analyses were generally robust, and no significant evidence of publication bias was found; the trim-and-fill method also indicated that no additional studies were needed. The dose–response results showed a nonlinear pattern, with the model indicating that the optimal cumulative dose was approximately 21.3 h (recommended dose range: 19.8–22.8 h), corresponding to an exploratory prescription of two 40-min sessions per week over approximately 15 weeks. Due to insufficient reporting of exercise intensity and adherence in the included primary studies, the “21.3-h” optimal dose proposed in this study should be considered an exploratory reference value for clinical practice rather than an absolute prescriptive standard. Overall, table tennis exercise can be a useful supplement for enhancing visual function in school settings, but its application should be carefully considered, given the study’s quality and variability.

### Limitations

Despite efforts to ensure methodological rigor, caution is necessary when interpreting the study’s limitations. First, there was considerable heterogeneity among the included studies, which may have impacted the accuracy of the pooled estimates. Second, the overall risk of bias was relatively high, primarily due to issues with randomization and deviations from the intended interventions (D1/D2), as well as inadequate reporting of missing outcome data and outcome measurement (D3/D4). Third, there was considerable variation in the intervention prescriptions, and the measurement tools were not completely consistent across studies, which may have decreased comparability. Fourth, although the dose–response model identified an optimal cumulative dose and a recommended range, its posterior uncertainty interval was relatively wide, suggesting considerable uncertainty at higher doses. Moreover, insufficiently monitored intervention adherence may also have affected the shape of the dose–response curve. Lastly, all included studies were conducted in China, so the applicability of the findings should be interpreted with caution.

## Data Availability

The original contributions presented in the study are included in the article/Supplementary material, further inquiries can be directed to the corresponding author.
