# Supplementary material for: Effects of table tennis on vision in children and adolescents: a multilevel dose–response meta-analysis
Source: Front Med (Lausanne). 2026 May 28;13:1829768. doi: 10.3389/fmed.2026.1829768 (PMC13253264; doi:10.3389/fmed.2026.1829768)
Supplement: Supplementary file 1 [file Supplementary_file_1.docx]

**Supplementary document Retrieval Strategy**

**1. PubMed**

(

("Racquet Sports"[Mesh] OR "Sports"[Mesh] OR "Exercise"[Mesh])

AND

("table tennis"[tiab] OR table-tennis[tiab] OR "ping pong"[tiab] OR ping-pong[tiab] OR pingpong[tiab] OR "table tennis player*"[tiab])

)

AND

("Child"[Mesh] OR "Adolescent"[Mesh] OR "Students"[Mesh] OR child*[tiab] OR adolescen*[tiab] OR teen*[tiab] OR youth*[tiab] OR pediatric*[tiab] OR paediatric*[tiab] OR "school-age"[tiab] OR schoolchild*[tiab] OR "school student*"[tiab])

AND

(

"Vision, Ocular"[Mesh] OR "Visual Acuity"[Mesh] OR "Myopia"[Mesh] OR "Refractive Errors"[Mesh]

OR vision[tiab] OR eyesight[tiab] OR "visual acuity"[tiab] OR myopia[tiab] OR nearsighted*[tiab]

OR "refractive error*"[tiab] OR "spherical equivalent"[tiab] OR diopter*[tiab]

OR accommodation[tiab] OR convergence[tiab] OR "binocular vision"[tiab] OR stereopsis[tiab]

)

AND

("Randomized Controlled Trial"[pt] OR randomized[tiab] OR randomised[tiab] OR randomization[tiab] OR randomly[tiab] OR "clinical trial"[tiab] OR "controlled trial"[tiab] OR "control group"[tiab] OR RCT[tiab])

NOT

("Review"[pt] OR "Case Reports"[pt] OR "Comment"[pt] OR "Editorial"[pt])

**2. Web of Science**

(TS=("table tennis" OR table-tennis OR "ping pong" OR ping-pong OR pingpong))

AND

(TS=(child* OR adolescen* OR teen* OR youth* OR pediatric* OR paediatric* OR schoolchild* OR "school student*"))

AND

(TS=(vision OR "visual acuity" OR eyesight OR myopia OR nearsighted* OR "refractive error*" OR "refractive status" OR "spherical equivalent" OR diopter* OR "binocular vision" OR stereopsis OR accommodation OR convergence))

AND

(TS=("randomized controlled trial" OR "randomised controlled trial" OR randomized OR randomised OR RCT OR trial OR "controlled trial" OR "control group"))

Timespan: All years to 2026-2-11.

**3. PsycINFO**

1. exp Racquet Sports/ or exp Table Tennis/ or ( "table tennis" or table-tennis or "ping pong" or ping-pong or pingpong ).ti,ab,id.

2. exp Children/ or exp Adolescents/ or exp Students/ or ( child* or adolescen* or teen* or youth* or pediatric* or paediatric* or schoolchild* or "school student*" ).ti,ab,id.

3. exp Vision/ or exp Visual Acuity/ or exp Myopia/ or exp Refractive Errors/ or ( vision or "visual acuity" or eyesight or myopia or nearsighted* or "refractive error*" or "spherical equivalent" or diopter* or accommodation or convergence or "binocular vision" or stereopsis ).ti,ab,id.

4. exp "Randomized Controlled Trials"/ or ( randomized or randomised or randomization or "random allocation" or "control group" or RCT or "clinical trial" or "controlled trial" ).ti,ab,id.

5. 1 AND 2 AND 3 AND 4

6. limit 5 to (english or chinese language)

**4. Cochrane Library**

#1 [mh "Racquet Sports"] OR ("table tennis" or table-tennis or "ping pong" or ping-pong or pingpong):ti,ab,kw

#2 [mh "Child"] OR [mh "Adolescent"] OR [mh "Students"] OR (child* or adolescen* or teen* or youth* or schoolchild* or "school student*"):ti,ab,kw

#3 [mh "Vision, Ocular"] OR [mh "Visual Acuity"] OR [mh "Myopia"] OR [mh "Refractive Errors"]

OR (vision or "visual acuity" or eyesight or myopia or nearsighted* or "refractive error*" or "spherical equivalent" or diopter* or stereopsis or accommodation or convergence or "binocular vision"):ti,ab,kw

#4 (randomized or randomised or randomization or "control group" or RCT or trial or "controlled trial"):ti,ab,kw OR [mh "Randomized Controlled Trials"]

#5 #1 AND #2 AND #3 AND #4

Publication Date to 2026 2-11

**5. CNKI**

SU=(乒乓球 OR 乒乓球运动 OR 乒乓运动 OR "Table Tennis" OR "Ping Pong")

AND

SU=(儿童 OR 青少年 OR 学生 OR 学龄儿童 OR 中小学生 OR 小学生 OR 初中生 OR 高中生)

AND

SU=(视力 OR 视敏度 OR 视力变化 OR 视力改善 OR 裸眼视力 OR 矫正视力 OR 屈光度 OR 屈光不正 OR 近视 OR 球镜等效 OR SE OR 双眼视功能 OR 立体视 OR 调节 OR 集合)

AND

SU=(随机对照试验 OR 随机 OR 对照 OR RCT OR 临床试验)


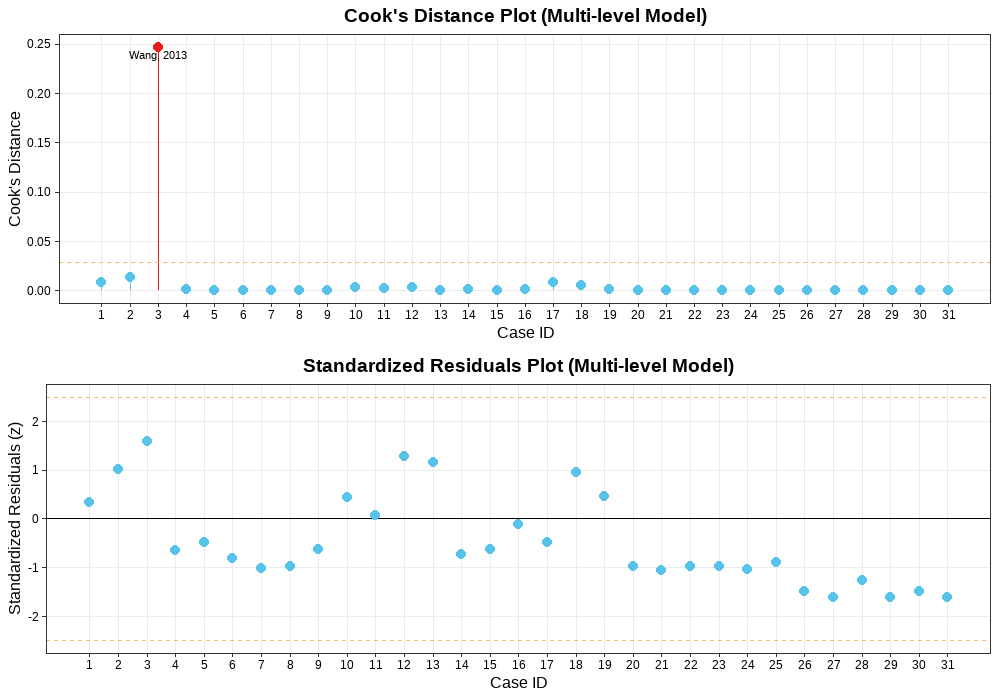


**Standardized Residuals and Cook's Distance Threshold Plot (Second Time)**


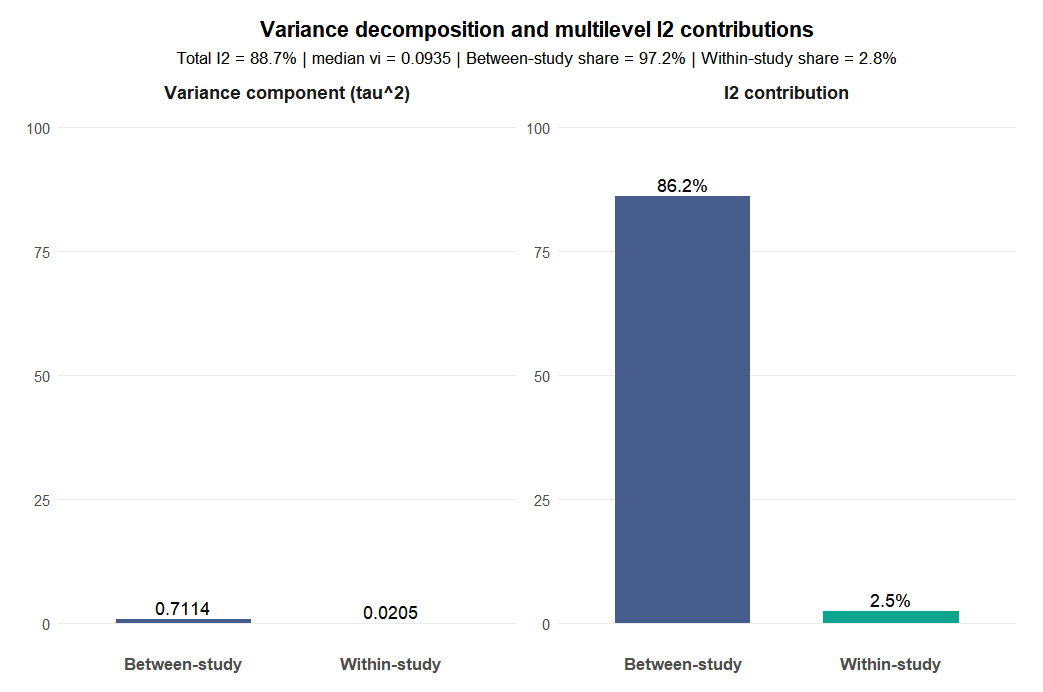


**Variance Decomposition Plot (Before Outlier Elimination)**


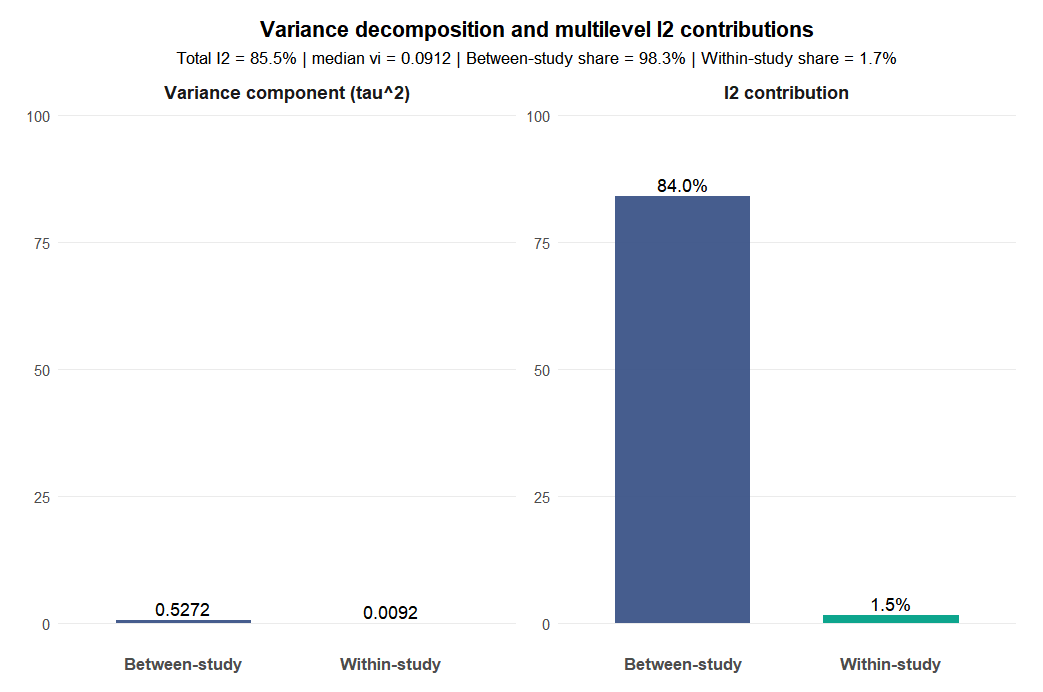


**Variance Decomposition Plot (After Outlier Removal)**


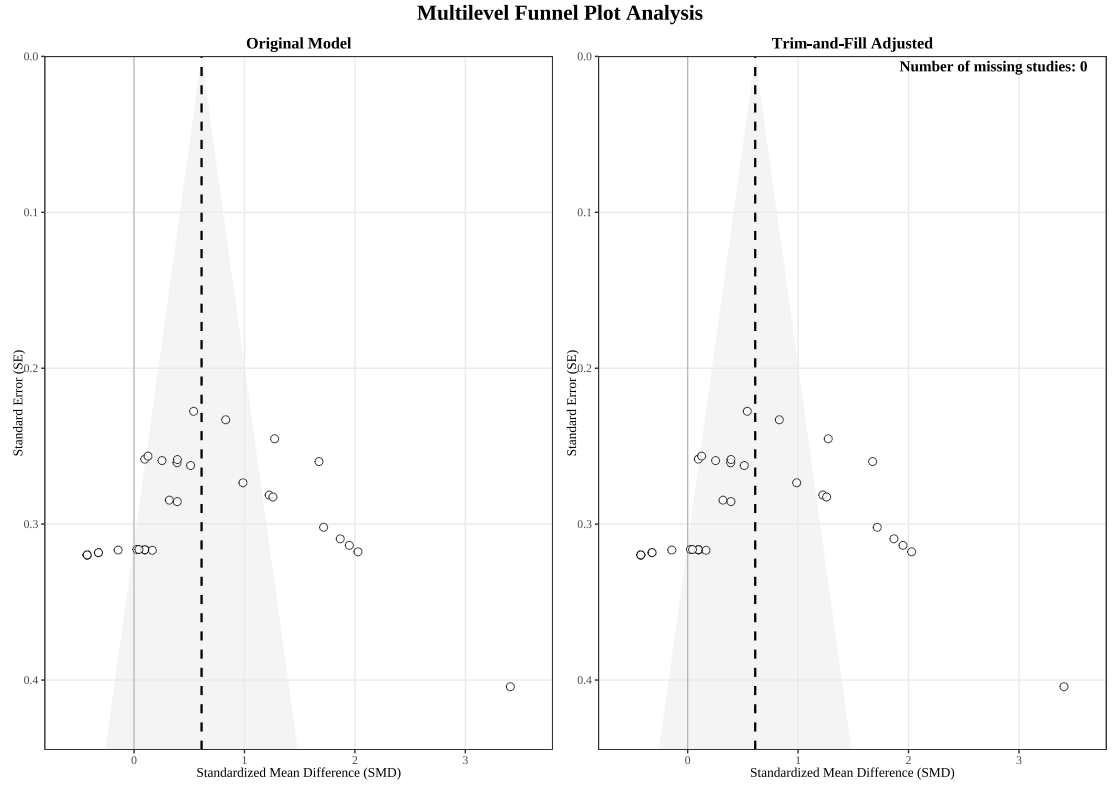


**Trim-and-Fill Plot**


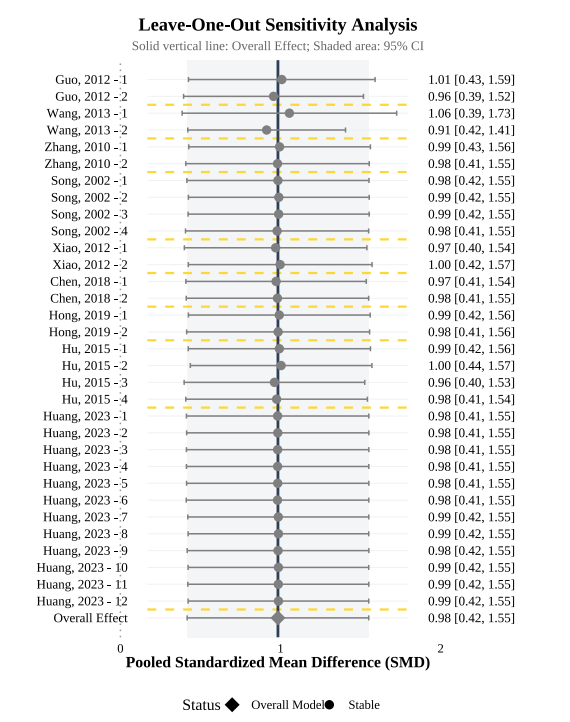


**Sensitivity Analysis Funnel Chart**


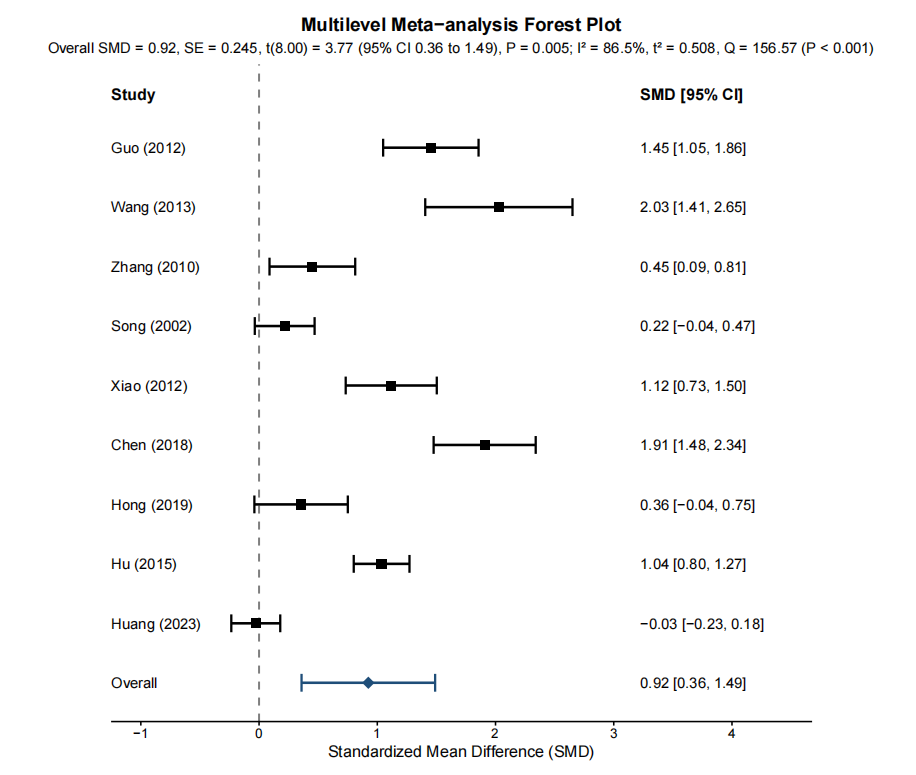


**Forest plot of the multilevel meta-analysis with small-sample corrected robust variance estimation**


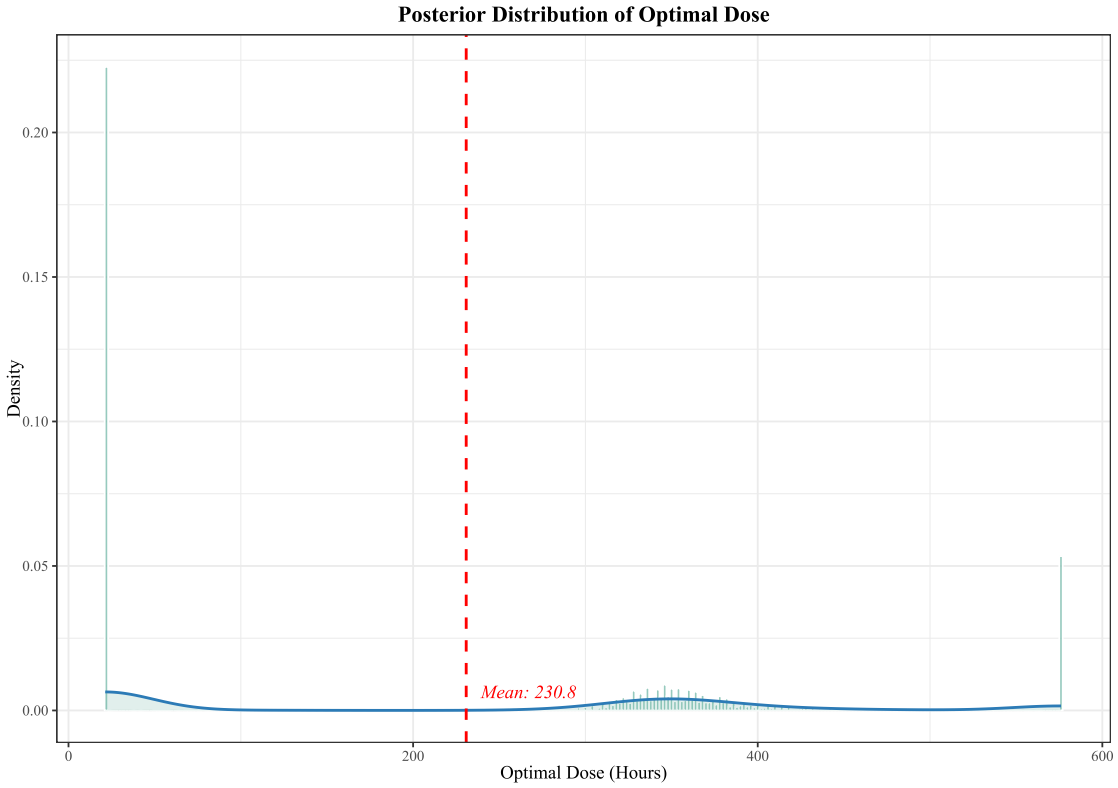


**Posterior Optimal Dose Distribution Plot**


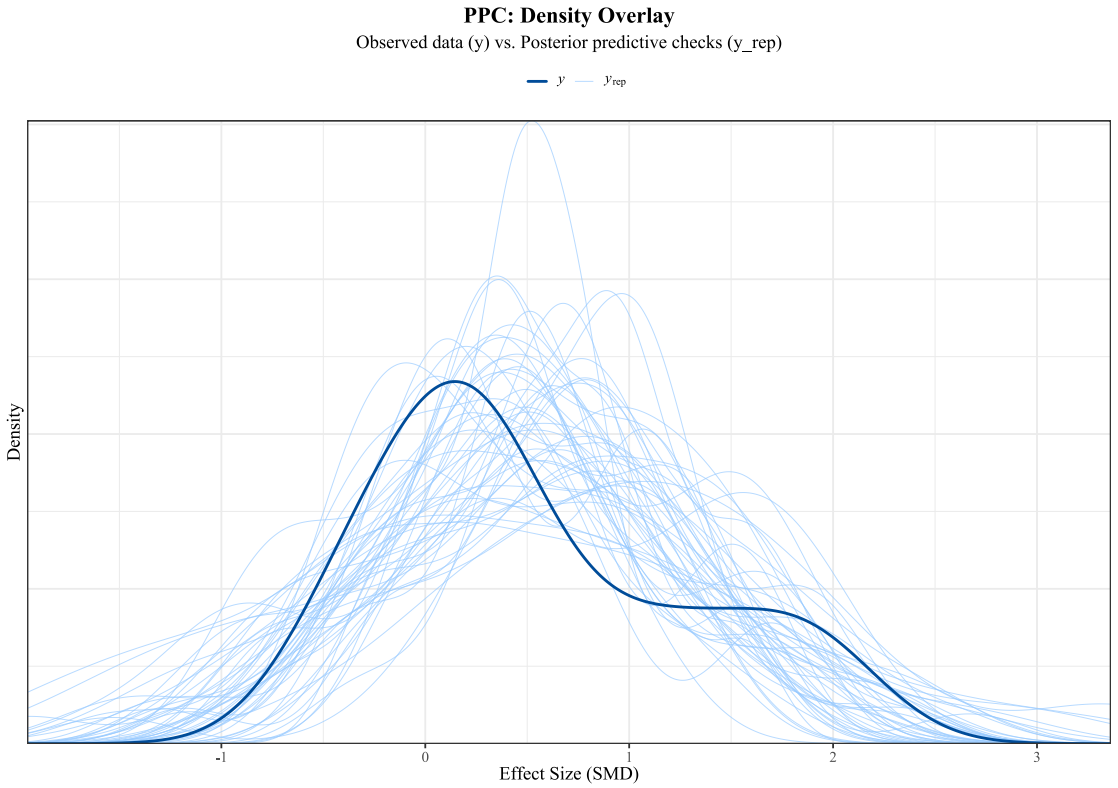


**Global Density Overlay (Observed yi vs. multiple yrep densities)**


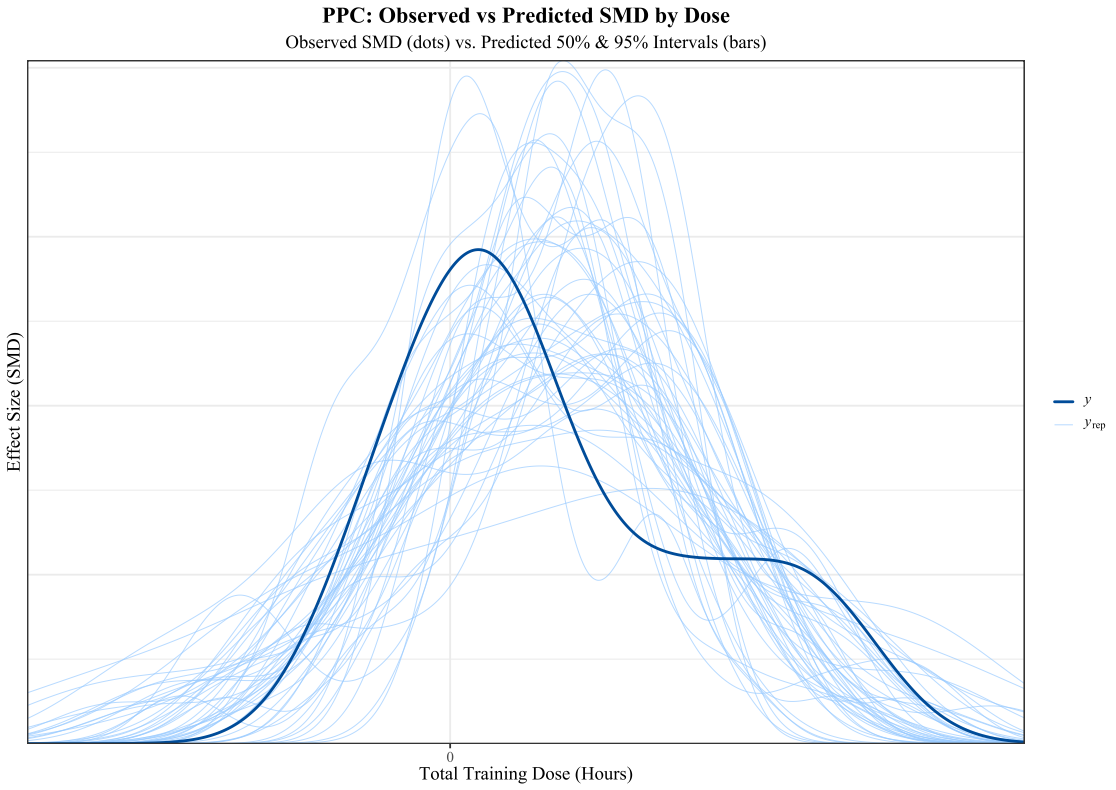


**Interval Plot: Showing posterior predictive intervals for each observation and plotting observed values**


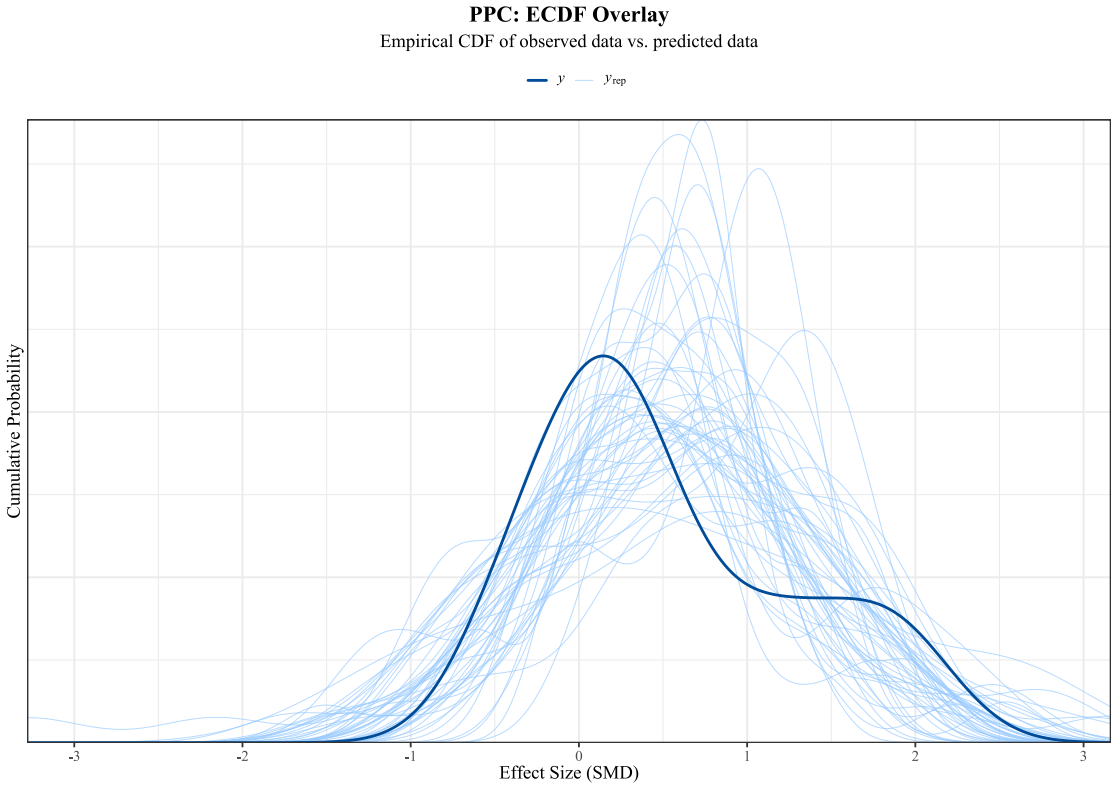


**ECDF Comparison**


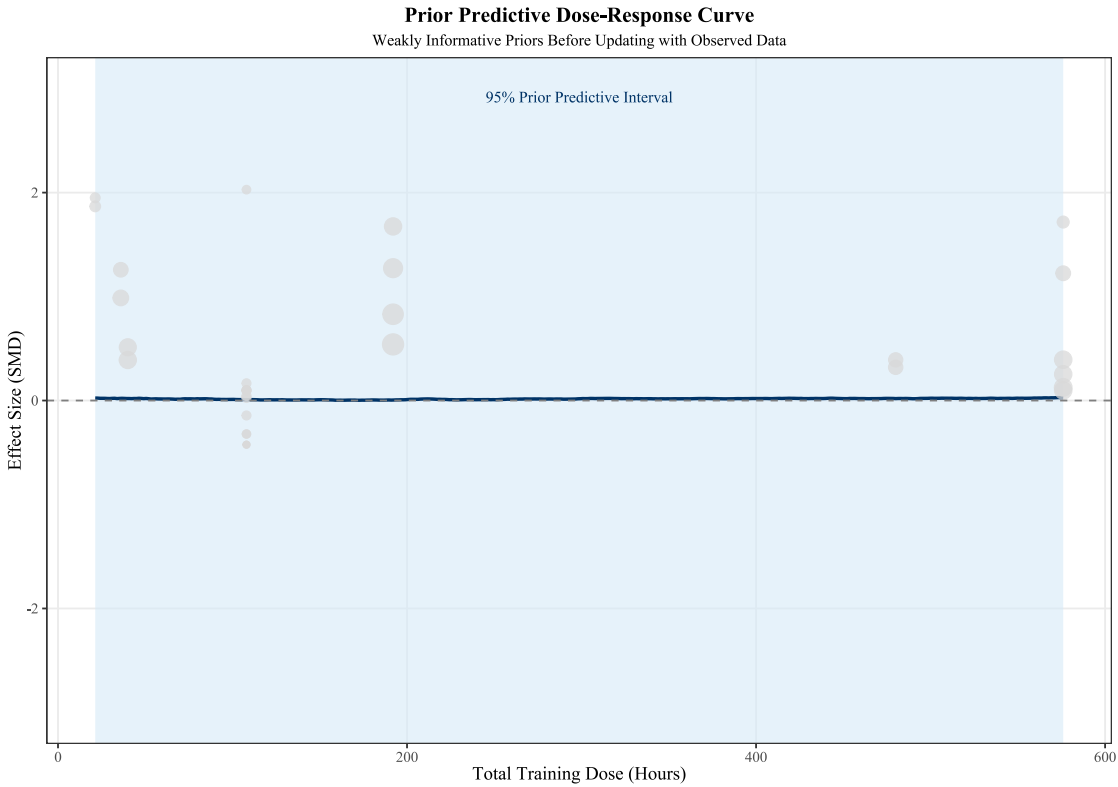


**Prior Interval Distribution Diagram**


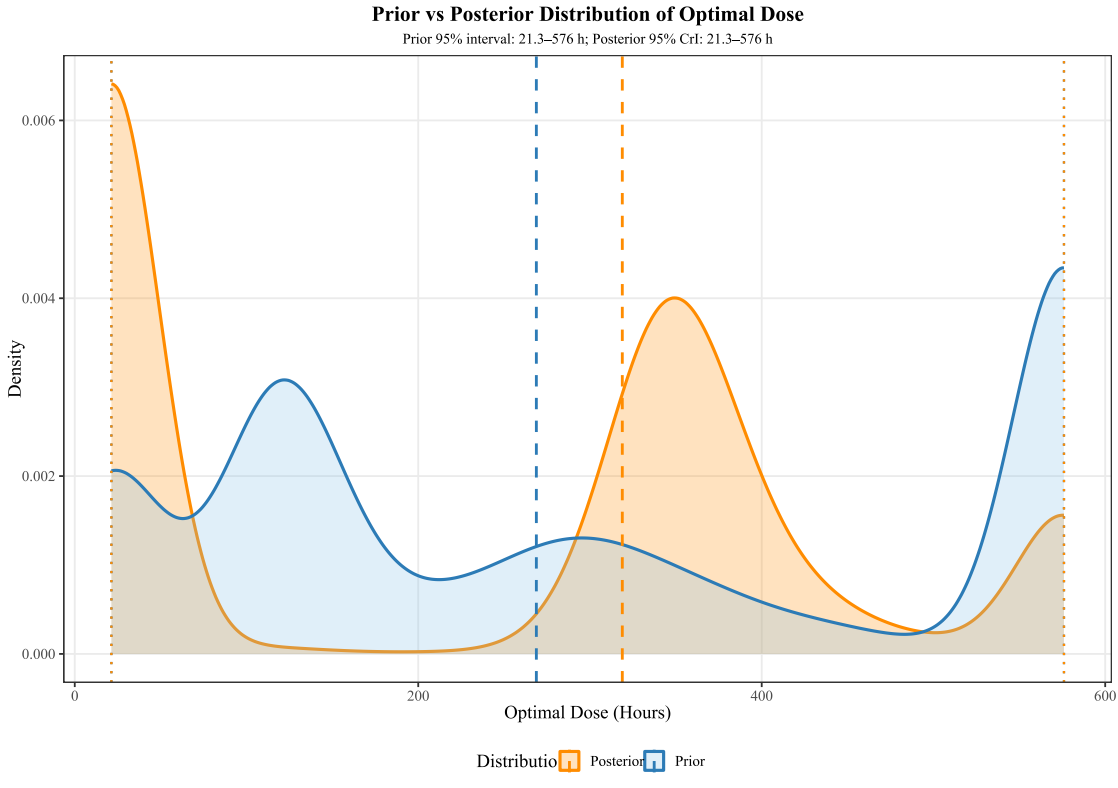


**Comparison Chart of Prior Interval and Posterior Interval**

**Inter-Rater Agreement Results for Each ROB2 Domain**

The table below displays the simple agreement rate, Cohen's Kappa, and Weighted Kappa calculated separately for each domain (D1-D5).

| Domain | Simple Agreement Rate | Cohen’s Kappa | Weighted Kappa | Strength of Agreement (Cohen's Kappa) |
| --- | --- | --- | --- | --- |
| D1  (Randomization process) | 66.7% (6/9) | 0.481 | 0.542 | Moderate |
| D2  (Deviations from intended interventions) | 66.7% (6/9) | 0.000 | 0.000 | Slight |
| D3  (Missing outcome data) | 66.7% (6/9) | 0.413 | 0.491 | Moderate |
| D4  (Measurement of the outcome) | 88.9% (8/9) | 0.000 | 0.000 | Slight |
| D5  (Selection of the reported result) | 100% (9/9) | 1.000 | 1.000 | Perfect/Almost Perfect |

**Note:** The inter-rater agreement between the two reviewers for each ROB2 domain was assessed. The Simple Agreement Rate​ was calculated as the percentage of studies for which both reviewers assigned identical judgments (Low, Some concerns, or High). Cohen's Kappa (κ)​ statistic, which accounts for chance agreement, was calculated from 3x3 contingency tables. Weighted Kappa​ was also computed using linear weights (0, 0.5, 1) to grant partial credit for adjacent rating disagreements (e.g., Low vs. Some concerns). The strength of agreement based on Cohen's Kappa was interpreted as follows: 0.81–1.00 = "Perfect/Almost Perfect", 0.61–0.80 = "Substantial/Good", 0.41–0.60 = "Moderate", 0.21–0.40 = "Fair", 0.00–0.20 = "Slight", and <0.00 = "Poor".

**GRADE Summary**

| GRADE domain | Judgment | Rationale |
| --- | --- | --- |
| Risk of Bias | Very serious → downgrade 2 levels | Based on the Final ROB2 we completed, 5/9 RCTs were rated Overall=High and the remaining 4/9 as Some concerns. The main concerns were concentrated in D1 (randomization process) and D2 (deviations from intended interventions), including insufficient reporting of sequence generation/allocation concealment and limited reporting of adherence, contamination control, and ITT analyses. Therefore, the certainty was downgraded by 2 levels for risk of bias. |
| Inconsistency | Serious → downgrade 1 level | Although sensitivity analyses suggested that the pooled effect remained statistically significant after removing one influential effect size, substantial heterogeneity remained. The heterogeneity may be related to differences in intervention duration, session frequency, comparator type, baseline visual status, and outcome assessment tools. Therefore, inconsistency was judged as serious. |
| Indirectness | Not serious | The included studies matched the intended PICO: children/adolescents, table tennis intervention, comparator conditions (e.g., usual care/physical education/no intervention/other exercise), and standardized measures of visual acuity. Therefore, no downgrade was applied for indirectness. |
| Imprecision | Not serious (temporary; no downgrade applied) | After removing the influential outlier, the pooled effect estimate was statistically significant and the 95% CI did not cross the null (SMD=0.91, 95% CI: 0.42–1.41; P<0.001), suggesting a clinically meaningful improvement. Although the number of trials was limited and individual study sample sizes were modest (typically 40–80 participants), the confidence interval remained away from no effect; therefore, no downgrade was applied for imprecision (provisional). |
| Publication Bias | Undetected (provisional) | Egger’s regression test did not indicate significant small-study effects (t=0.903, P=0.375; after removing the influential outlier: P=0.705). Trim-and-fill analysis suggested no missing studies were required. Taken together, publication bias was recorded as Undetected (provisional) and no downgrade was applied. |
| Overall certainty of evidence | Very Low | Evidence from RCTs starts as High. It was downgraded by 2 levels to Low due to a Very serious Risk of Bias. No additional downgrading was applied for other domains (provisional, based on the analyses reported above). |

**Subgroup Analysis of Table Tennis Sport on visual acuity-related outcomes in Children and Adolescents**

| Dimensionality | sort | K | I^2^ | Effect model | SMD and 95%CI | P | P-interaction​​​ |
| --- | --- | --- | --- | --- | --- | --- | --- |
| Intervention frequency/week |  |  |  |  |  |  | < 0.001 |
|  | 3 | 16 | 82.1% | Random | 0.48(0.13, 0.84) | 0.008 |  |
|  | 4 | 12 | 74.8% | Random | 0.61(0.30, 0.93) | < 0.001 |  |
|  | 2 | 2 | 0.0% | Random | 1.91(1.47, 2.34) | < 0.001 |  |
| Intervention cycle/week |  |  |  |  |  |  | 0.013 |
|  | > 24 | 12 | 79.0% | Random | 0.73(0.40, 1.06) | < 0.001 |  |
|  | 24 | 10 | 77.3% | Random | 0.18(-0.24, 0.59) | 0.406 |  |
|  | < 24 | 6 | 80.8% | Random | 1.14(0.62, 1.67) | < 0.001 |  |
| Intervention time/minute |  |  |  |  |  |  | 0.036 |
|  | > 90 | 4 | 81.0% | Random | 0.91(0.25, 1.57) | 0.007 |  |
|  | 90 | 18 | 80.5% | Random | 0.40(0.10, 0.70) | 0.010 |  |
|  | < 90 | 6 | 80.8% | Random | 1.14(0.62, 1.67) | < 0.001 |  |
| Total Intervention |  |  |  |  |  |  | 0.013 |
|  | > 108 | 12 | 79.0% | Random | 0.73(0.40, 1.06) | < 0.001 |  |
|  | 108 | 10 | 77.3% | Random | 0.18(-0.24, 0.59) | 0.408 |  |
|  | < 108 | 6 | 80.8% | Random | 1.14(0.62, 1.67) | <0.001 |  |
| Subject type |  |  |  |  |  |  | 0.001 |
|  | Adolescents | 2 | 28.7% | Random | 1.46(0.98, 1.94) | < 0.001 |  |
|  | Children | 26 | 81.7% | Random | 0.57(0.30, 0.83) | < 0.001 |  |
| Intervention mode2 |  |  |  |  |  |  | 0.005 |
|  | NIC | 2 | 28.7% | Random | 1.46(0.98, 1.94) | < 0.001 |  |
|  | UPEC | 13 | 83.1% | Random | 0.64(0.25, 1.04) | 0.001 |  |
|  | OPC | 13 | 81.8% | Random | 0.49(0.13, 0.85) | 0.008 |  |
| Eye assessed |  |  |  |  |  |  | 0.382 |
|  | OS | 12 | 80.4% | Random | 0.71(0.34, 1.07) | < 0.001 |  |
|  | OD | 15 | 81.5% | Random | 0.48(0.13, 0.83) | 0.007 |  |
| Age |  |  |  |  |  |  | < 0.001 |
|  | > 11 | 3 | 45.6% | Random | 1.64(1.17, 2.10) | < 0.001 |  |
|  | < 11 | 14 | 79.8% | Random | 0.87(0.55, 1.19) | < 0.001 |  |
|  | 11 | 11 | 0% | Random | 0.04(-0.13, 0.22) | 0.628 |  |
| Visual acuity status |  |  |  |  |  |  | 0.118 |
|  | Pseudomyopia | 4 | 54.9% | Random | 0.78(0.38, 1.17) | < 0.001 |  |
|  | Myopia | 13 | 81.4% | Random | 0.33(-0.07, 0.73) | 0.103 |  |

**Note:​** SMD, Standardized Mean Difference; statistical significance is defined as a 95% Confidence Interval (95% CI) that does not include zero. P-interaction is the p-value for tests of subgroup differences. Intervention modes2: NIC, No-intervention control; UPEC, Usual physical education class; OPC, Outdoor physical activity. Eye assessed: OS, oculus sinister; OD, oculus dexter.

**Sorting Table of Table Tennis Intervention Intensity in Original Studies**

| Study | Protocol (session duration, frequency/week, total duration) | Objective intensity indicators reported | Intensity information extracted from the original study |
| --- | --- | --- | --- |
| Guo et al., 2012 | 120 min/session, 3×/week, 96 weeks | Not reported | The study reported long-term regular table tennis training and assessed agility-related outcomes, including whole-body agility, upper-limb object control, and lower-limb footwork-related performance. However, no heart rate, RPE, METs, exercise density, stroke frequency, or detailed training load was reported. |
| Wang & Liu, 2013 | 90 min/session, 3×/week, 24 weeks | Not reported | The study explicitly described table tennis as a sport with a moderate amount of exercise and stated that its physiological load was mainly characterized by moderate-intensity aerobic metabolism. |
| Zhang & Li, 2010 | 60 min/session, 4×/week, 10 weeks | Not reported | The study reported training frequency, session duration, and total intervention period, but did not describe the detailed training structure or physiological intensity. |
| Song et al., 2002 | 90 min/session, 4×/week, 96 weeks | Not reported | The study compared students with long-term table tennis training experience and non-trained students, and also followed myopic children after continuous training. However, the detailed training structure, exercise density, and physiological intensity were not reported. |
| Xiao, 2012 | 45 min/session, 3×/week, 16 weeks | Not reported | The study focused on comparing table tennis and middle-distance running for pseudomyopia improvement. It did not provide objective intensity indicators or detailed table tennis training contents. |
| Chen et al., 2018 | 40 min/session, 2×/week, 16 weeks | Not reported | The exercise prescription table classified different training modules by intensity: multi-ball receiving/hitting and visual reaction drills were described as moderate intensity; left–right/forward–backward catching, speed-varied catching, ball throwing, attention games, relay dribbling, and racket-control drills were described as low intensity; shadow swing, rope skipping, and integrated footwork training were described as high intensity. |
| Hong, 2019 | 120 min/session, 3×/week, 80 weeks | Not reported | The study reported the intervention frequency, session duration, and total intervention period, but did not describe whether the intervention consisted of basic hitting, footwork drills, multi-ball practice, or match play. |
| Hu, 2015 | 90 min/session, 4×/week, 32 weeks | Not reported | The study reported weekly frequency and session duration, but did not provide detailed content of the table tennis training group or physiological intensity indicators. |
| Huang et al., 2023 | 90 min/session, 3×/week, 24 weeks | Not reported | The study reported the intervention frequency, class duration, and total intervention period, and described the sports programs as professionally designed. However, no heart rate, RPE, METs, exercise density, stroke frequency, or detailed training intensity was reported. |
